# Opioid Cap Laws and Opioid Prescriptions After Total Joint Replacements in Older Adults

**DOI:** 10.1001/jamanetworkopen.2025.4448

**Published:** 2025-04-09

**Authors:** Caroline P. Thirukumaran, Derek T. Schloemann, Jalpa A. Doshi, Kevin A. Fiscella, Benjamin F. Ricciardi, Meredith B. Rosenthal

**Affiliations:** 1 Department of Orthopaedic Surgery, Feinberg School of Medicine, Northwestern University, Chicago, Illinois; 2 Department of Orthopaedics and Physical Performance, University of Rochester, Rochester, New York; 3 Division of General Internal Medicine, University of Pennsylvania Perelman School of Medicine, Philadelphia; 4 Department of Family Medicine, University of Rochester, Rochester, New York; 5 Department of Health Policy and Management, Harvard T.H. Chan School of Public Health, Boston, Massachusetts

## Abstract

**Importance:**

Despite the opioid crisis and a growing call for minimizing opioid use, opioids remain an important part of postoperative pain management, with more than 80% of patients filling at least 1 opioid prescription following total joint replacements (TJRs). Little is known as to whether state laws that restrict or cap opioids for acute pain reduce post-TJR opioid use.

**Objective:**

To evaluate the association of an opioid cap law in New York (Section 3331) with post-TJR opioid prescribing.

**Design, Setting, and Participants:**

This cohort study analyzed Medicare data from 2014 to 2019 for New York and California (control state). Participants were Medicare beneficiaries who underwent elective TJRs before (April 2014 to June 2016) or after (August 2016 to September 2019) Section 3331 implementation. Data were analyzed from June 2023 to August 2024.

**Exposure:**

Implementation of New York Section 3331 in July 2016.

**Main Outcomes and Measures:**

The primary end point was total morphine milligram equivalents (MMEs) filled from discharge to day 7, days 8 to 30, and days 31 to 90 after TJR. Key independent variables were legislation phase (before or after Section 3331 implementation), treatment or control state, and the interactions between these 2 variables. Difference-in-differences regression models were used to assess the association of interest.

**Results:**

The pre–Section 3331 cohort included 32 253 TJR encounters among 31 028 patients, of whom 9924 (31.98%) underwent TJRs in New York hospitals. The mean (SD) age of the cohort was 73.43 (5.49) years; 19 442 encounters (60.28%) were among females. The estimated change in total MMEs filled in the 7-day post-TJR period after vs before Section 3331 implementation was −135.08 (95% CI, −146.62 to −123.53; *P* < .001) in California and −178.00 (95% CI, −191.98 to −164.02; *P* < .001) in New York, resulting in a Section 3331–associated change of −42.92 MMEs (95% CI, −61.04 to −24.80 MMEs; *P* < .001) in New York compared with California. Section 3331 was not associated with statistically significant changes in total MMEs filled in the 8 to 30–day and 31 to 90–day post-TJR periods.

**Conclusions and Relevance:**

The findings of this retrospective cohort study of TJRs among Medicare beneficiaries suggest that New York Section 3331 achieved its intended objective, as it was associated with reduced opioid fills in the immediate 7-day post-TJR period in New York compared with California. Additional refinements may further reduce opioid prescribing in New York, and these findings may serve as a foundation for refining laws in other states that may not achieve their intended targets or have not implemented similar laws.

## Key Points

**Question:**

Was a 2016 opioid prescribing cap law in New York associated with changes in opioid prescribing for pain following total joint replacements (TJRs)?

**Findings:**

In this cohort study of 31 028 Medicare beneficiaries, the New York law was associated with a decrease in total quantity of opioids filled in the immediate 7 days following TJRs in New York compared with California (control state). This reduction likely was attributable to decreases in quantity of opioids filled per prescription and duration of opioid prescriptions in New York compared with California.

**Meaning:**

The findings suggest that the New York opioid restriction law was associated with reductions in opioid quantities in the immediate TJR postoperative period.

## Teaser

This cohort study investigates whether a New York state law capping opioid prescribing was associated with a decrease in the quantity of opioids filled following total joint replacements among Medicare beneficiaries.

## Introduction

Elective total hip and knee replacements (collectively, total joint replacements [TJRs]) are associated with reduced pain, improved physical function, and enhanced health-related quality of life for patients with advanced osteoarthritis.^1,2,3^ Adequate post-TJR pain control is a key marker of successful surgery,^4^ and inadequate control is associated with impaired recovery, resulting in delayed or unmet physical therapy milestones, increased health services use, and reduced quality of life.^5,6^ Despite the opioid crisis and a growing call for minimizing opioid use while increasing multimodal analgesia (a combination of opioid and nonopioid analgesics),^7,8,9,10,11^ opioids remain an important component of post-TJR pain relief, with more than 80% of Medicare beneficiaries filling at least 1 opioid prescription in the immediate post-TJR period.^12,13^ Opioids are often prescribed in excess of pain needs, with an average of 218 morphine milligram equivalents (MMEs) of opioids (eg, twenty-nine 5-mg oxycodone pills) remaining unused per patient,^14,15^ potentially contributing to long-term dependence and misuse.

State opioid cap laws (SOCLs) have been implemented by at least 39 states to restrict opioid prescribing for acute pain, thereby reducing (1) prolonged treatment with opioids that increase addiction risk, (2) unused opioids, and (3) opioids that are likely to be diverted for misuse.^16,17,18^ New York state, which has a high TJR volume, implemented its SOCL (Section 3331) on July 22, 2016.^19^ This law limits the initial prescription of opioids for acute pain to 7 days, applies only to the initial opioid prescription, and does not allow prescribers to exceed the duration limit using their clinical judgment. The only study to our knowledge that has examined the association of Section 3331 with post-TJR opioid use found that Section 3331 was associated with a significant decrease in post-TJR opioid prescribing.^20^ However, the validity of findings from that study is limited by the lack of a control group and limited generalizability to hospitals across the country. Furthermore, besides a few TJR-focused single-center studies that examined SOCLs in other states and 1 national study,^15,21,22,23,24,25,26,27^ there is little generalizable evidence about how SOCLs have influenced post-TJR opioid prescribing, especially in New York.

Our objective was to assess the association of Section 3331 with opioid prescribing for post-TJR pain in New York compared with the control state of California, which did not have a similar SOCL in effect during the study period. Our work is critical for understanding whether SOCLs may be effective in limiting the prescription of opioids for one of the highest-priority procedures for older adults, for identifying refinements that can be introduced in SOCL design, and for supporting the implementation of SOCLs in 11 states and the District of Columbia that currently do not have SOCLs.

## Methods

This cohort study was exempted from review and informed consent was waived by the Research Subject Review Board of the University of Rochester, New York, because this was a retrospective analysis of Medicare data that were deidentified for research purposes and there was minimal risk to study participants. We followed the Strengthening the Reporting of Observational Studies in Epidemiology (STROBE) reporting guideline for observational studies.^28^

### Data Sources and Study Cohort

We used Medicare Provider Analysis and Review (MedPAR) files from 2014 to 2019,^29^ which include claims for inpatient stays, to identify fee-for-service Medicare beneficiaries who underwent inpatient total hip or knee replacements^30^ (eMethods 1 in Supplement 1) in New York and California. We linked these extracts to the Medicare Master Beneficiary Summary File (MBSF), which includes enrollment details for all Medicare beneficiaries, and other MBSF files that include beneficiaries’ chronic conditions data.^31,32,33^ We limited the cohort to beneficiaries covered by the Medicare fee-for-service (Parts A and B) and prescription drug (Part D) programs for the entire calendar year, TJRs performed for elective reasons,^30^ and patients discharged to home (because medication claims from patients in skilled nursing or inpatient rehabilitation facilities are not included in Medicare’s prescription claims data).^34^ We also used other criteria detailed in eMethods 2 in Supplement 1.

We linked this cohort to the Medicare Part D event file to identify prescription claims initiated 90 days before admission and 90 days after discharge from the inpatient TJR encounter.^35^ The event file includes claims for outpatient prescription drugs filled by Medicare beneficiaries with Part D coverage. We linked this extract to the Medicare Impact File to obtain hospital characteristics.^36^

Race and ethnicity were determined from the MBSF, which obtains data from Social Security Administration records,^37^ and were included in the analysis to control for race- and ethnicity-associated variability in the outcome. Categories were Asian; Black; Hispanic; North American Native, unknown, or other (grouped to comply with Medicare’s cell size policy); and White.

### Opioid Prescribing End Points

The primary end points were the total MMEs filled in the period from the date of discharge to 7 days after discharge (7-day post-TJR period), 8 to 30 days after discharge, and 31 to 90 days after discharge from a TJR stay. These cutoffs were chosen to coincide with the duration limit of 7 days set by Section 3331 and key clinical practice milestones.

Secondary end points were (1) MMEs per prescription and day, (2) likelihood of at least 1 opioid fill, (3) number of opioid fills, (4) likelihood of an opioid prescription longer than 7 days, and (5) days’ supply of opioids (total and per prescription). Of these, the primary outcome and secondary outcomes of MMEs per prescription and day, number of opioid fills, and days’ supply of opioids were modeled as continuous indicators; the other outcomes were modeled as binary indicators. End points were examined for all 3 periods except for likelihood of an opioid prescription longer than 7 days, which was examined only for the 7-day postoperative period.

### Key Independent Variables and Covariates

The first key independent variable was legislation phase: before Section 3331 (April 2014 to June 2016) and after Section 3331 (August 2016 to September 2019), modeled as a binary indicator. This specification excluded July 2016, which was the month in which Section 3331 was implemented. The second independent variable was treatment (New York) or control (California) state, modeled as a binary indicator, and the third was a statistical interaction between these 2 variables. We chose California as the control state because it did not have legislation limiting the prescription of opioids for acute pain until the start of 2020, providing us with the longest post–Section 3331 period, and because California had the highest state-level volume of TJRs (7%) according to the Medicare data. In multivariable analysis, regression models controlled for patient- and hospital-level covariates (eMethods 3 in Supplement 1).

### Statistical Analysis

We report means and SDs for continuous variables and frequencies and percentages for categorical variables. For multivariable analysis, we estimated multivariable hierarchical linear (MMEs, number of fills, and days’ supply) and logistic (likelihood of ≥1 opioid fill and opioid prescription for >7 days) regression models with difference-in-differences (DID) estimation, with hospital-level random effects (eMethods 4 in Supplement 1).^38^ DID is an econometric method commonly used for policy evaluation. For the DID estimates, we computed 2 differences. The first was the difference between the pre–Section 3331 and post–Section 3331 periods separately for New York and California, and the second was the difference between New York and California during these phases. We report adjusted estimated probabilities expressed as percentages and means.

Before estimating these models, we tested for parallel trends for the end points in New York compared with California before the implementation of Section 3331 to support the assumption that the counterfactual trend in New York would be similar to California in the absence of Section 3331 (eMethods 4 in Supplement 1). We tested this by estimating multivariable hierarchical linear or logistic regression models using data from the pre–Section 3331 period, in which the statistical interaction between the year quarter and the state variables was the key estimate of interest. In case of a violation of this assumption,^39^ we adjusted for the differential linear trends in the main DID models.^40,41^ Data were analyzed from June 2023 to August 2024 using Stata/MP, version 17.0 for Unix (StataCorp LLC). Two-sided *P* < .05 was considered significant.

To test the robustness of our findings and how Section 3331 may have been associated with opioid prescribing for different subgroups, we conducted several sensitivity and secondary analyses. These are detailed in eMethods 5 in Supplement 1.

## Results

### Descriptive Statistics

The study included 85 572 Medicare beneficiaries and 93 527 TJR encounters. The pre–Section 3331 cohort included 32 253 encounters among 31 028 patients, of whom 9924 (31.98%) underwent TJRs in New York hospitals (Table 1). The mean (SD) age in the cohort was 73.43 (5.49) years; 19 442 encounters (60.28%) were among females, and 12 811 (39.72%) were among males. A total of 648 encounters (2.01%) were among Asian patients; 793 (2.46%), among Black patients; 749 (2.32%), among Hispanic patients; 1309 (4.06%), among North American Native patients or those with unknown or other race and ethnicity; and 28 754 (89.15%), among White patients. Descriptive statistics for the 356 included hospitals are in eTable 1 in Supplement 1.

**Table 1.** Descriptive Statistics of the Cohort in the Pre–Section 3331 Period From April 2014 to June 2016

Characteristic	Encounters^a^			*P* value^b^
	California (n = 21 963)	New York (n = 10 290)	Total (n = 32 253)	
Patients, No.	21 104	9924	31 028	NA
Hospitals, No.	237	119	356	NA
Age, mean (SD), y	73.44 (5.51)	73.41 (5.44)	73.43 (5.49)	.90
Sex				
Female	13 220 (60.19)	6222 (60.47)	19 442 (60.28)	.64
Male	8743 (39.81)	4068 (39.53)	12 811 (39.72)	
Race and ethnicity				
Asian	569 (2.59)	79 (0.77)	648 (2.01)	<.001
Black	407 (1.85)	386 (3.75)	793 (2.46)	
Hispanic	661 (3.01)	88 (0.86)	749 (2.32)	
North American Native, unknown or other^c^	911 (4.14)	398 (3.87)	1309 (4.06)	
White	19 415 (88.40)	9339 (90.76)	28 754 (89.15)	
Dual eligibility for Medicare and Medicaid	2394 (10.90)	690 (6.71)	3084 (9.56)	<.001
Surgery				
Hip replacement	7861 (35.79)	4690 (45.58)	12 551 (38.91)	<.001
Knee replacement	14 102 (64.21)	5600 (54.42)	19 702 (61.09)	<.001
Discharge destination				
Home with self-care	6662 (30.33)	2455 (23.86)	9117 (28.27)	<.001
Home with home health	15 301 (69.67)	7835 (76.14)	23 136 (71.73)	
Preoperative drug fills in 90 d before admission				
≥3 Opioid fills	2593 (11.81)	582 (5.66)	3175 (9.84)	<.001
≥3 Benzodiazepine fills	595 (2.71)	166 (1.61)	761 (2.36)	<.001
Elixhauser comorbidities^d^				
Mean (SD), No.	1.79 (1.37)	2.12 (1.38)	1.89 (1.38)	<.001
AIDS or HIV infection	NR^e^	NR^e^	NR^e^	.42
Blood loss anemia	108 (0.49)	34 (0.33)	142 (0.44)	.04
Cardiac arrhythmias	2911 (13.25)	1395 (13.56)	4306 (13.35)	.46
Chronic pulmonary disease	2727 (12.42)	1414 (13.74)	4141 (12.84)	.001
Coagulopathy	425 (1.94)	260 (2.53)	685 (2.12)	.001
Congestive heart failure	523 (2.38)	231 (2.24)	754 (2.34)	.45
Deficiency anemia	178 (0.81)	79 (0.77)	257 (0.80)	.69
Diabetes				
Complicated	346 (1.58)	145 (1.41)	491 (1.52)	.26
Uncomplicated	2955 (13.45)	1504 (14.62)	4459 (13.83)	.01
Fluid and electrolyte disorders	1287 (5.86)	1021 (9.92)	2308 (7.16)	<.001
Hypertension				
Complicated	1155 (5.26)	458 (4.45)	1613 (5.00)	.002
Uncomplicated	12 952 (58.97)	6539 (63.55)	19 491 (60.43)	<.001
Hypothyroidism	3846 (17.51)	1863 (18.10)	5709 (17.70)	.19
Liver disease	203 (0.92)	114 (1.11)	317 (0.98)	.12
Obesity	3237 (14.74)	3435 (33.38)	6672 (20.69)	<.001
Other neurological disorders	322 (1.47)	147 (1.43)	469 (1.45)	.79
Paralysis	NR^e^	NR^e^	NR^e^	.04
Peptic ulcer disease excluding bleeding	69 (0.31)	43 (0.42)	112 (0.35)	.14
Peripheral vascular disorders	451 (2.05)	260 (2.53)	711 (2.20)	.01
Pulmonary circulation disorders	109 (0.50)	93 (0.90)	202 (0.63)	<.001
Kidney failure	1149 (5.23)	463 (4.50)	1612 (5.00)	.01
Rheumatoid arthritis or collagen vascular diseases	961 (4.38)	404 (3.93)	1365 (4.23)	.06
Valvular disease	969 (4.41)	708 (6.88)	1677 (5.20)	<.001
Weight loss	50 (0.23)	26 (0.25)	76 (0.24)	.67
Other comorbidities				
Mental health conditions^f^	5852 (26.64)	2839 (27.59)	8691 (26.95)	.07
Alcohol use disorder^g^	396 (1.80)	163 (1.58)	559 (1.73)	.16
Tobacco use disorder^h^	910 (4.14)	551 (5.35)	1461 (4.53)	<.001

Abbreviations: NA, not applicable; NR, not reported.

^a^ Data are presented as number (percentage) of encounters unless otherwise indicated. California was the control state, and New York was the treatment state.

^b^ From χ^2^ or Kruskal-Wallis tests comparing the distribution of variables across patients in New York and California.

^c^ The counts of patients with race and ethnicity classified as North American Native, unknown, or other in the Medicare data were grouped to comply with Medicare’s cell size policy.

^d^ The list excludes lymphoma, metastatic cancer, solid tumor without metastasis, and drug abuse because patients with these conditions were excluded from the study cohort and psychoses, depression, and alcohol abuse because these were included in composites of mental health conditions and alcohol use disorder.

^e^ Values masked per Medicare’s cell size policy.

^f^ Included anxiety, bipolar disorder, depression, personality disorder, posttraumatic stress disorder, psychosis, schizophrenia, and other psychotic disorders defined using a combination of data in the Chronic Conditions file and Other Chronic or Potentially Disabling Conditions file in the Medicare Master Beneficiary Summary File and the Elixhauser algorithm.

^g^ Defined using a combination of data in the Other Chronic or Potentially Disabling Conditions file of the Medicare Master Beneficiary Summary File and the Elixhauser algorithm.

^h^ Defined using data in the Other Chronic or Potentially Disabling Conditions file of the Medicare Master Beneficiary Summary File.

### Unadjusted Trends

In the second quarter of 2014, patients filled a mean (SD) of 663.29 (482.12) MMEs in New York and 724.34 (861.04) MMEs in California during the 7-day post-TJR period. These measures declined to a mean (SD) of 595.31 (367.66) MMEs in New York and increased to 750.52 (670.56) MMEs in California in the third quarter of 2016, when Section 3331 was implemented in New York, and were 321.02 (207.63) MMEs in New York and 424.54 (297.45) MMEs in California at the end of the study period in the third quarter of 2019 (Figure). Similar trends continued in the periods from 8 to 30 days and 31 to 90 days after TJR.

**Figure.**
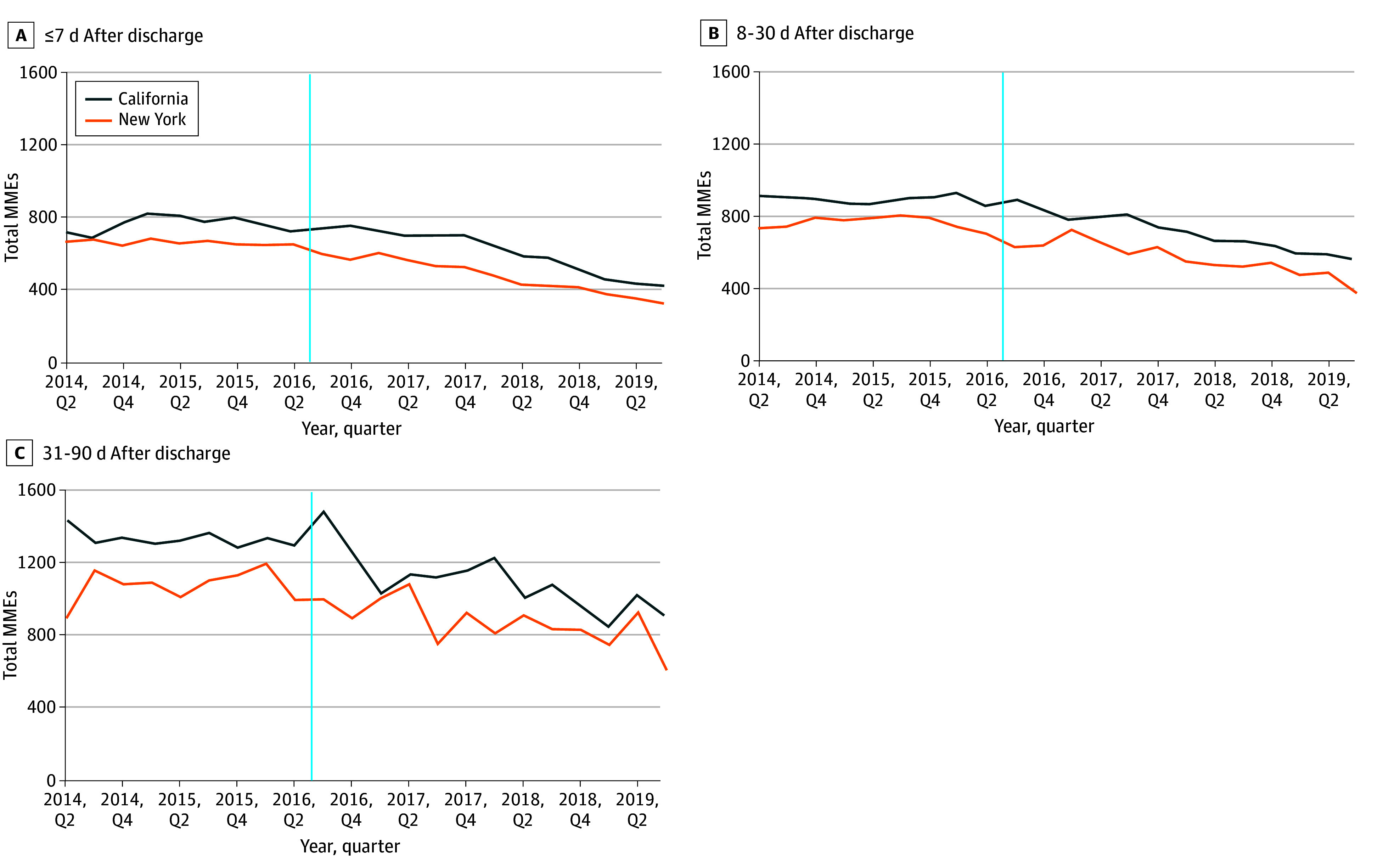
Unadjusted Trends in Mean Total Morphine Milligram Equivalents (MMEs) of Opioid Prescriptions Filled From April 2014 to September 2019 Vertical lines represent implementation of Section 3331 on July 22, 2016.

### Multivariable Analysis

#### MMEs

After Section 3331 was implemented, the estimated change in total MMEs filled in the 7-day post-TJR period was −135.08 (95% CI, −146.62 to −123.53; *P* < .001) in California and −178.00 (95% CI, −191.98 to −164.02; *P* < .001) in New York compared with before implementation, resulting in a Section 3331–associated change of −42.92 MMEs (95% CI, −61.04 to −24.08 MMEs; *P* < .001) in New York compared with California (Table 2 and eTable 2 in Supplement 1). Similarly, change in MMEs per prescription in the 7-day post-TJR period was −41.18 (95% CI, −48.45 to −33.91; *P* < .001) in California and −101.51 (95% CI, −110.31 to −92.70; *P* < .001) in New York after implementation compared with before, resulting in a Section 3331–associated change of −60.33 MMEs (95% CI, −71.74 to −48.91 MMEs; *P* < .001) per prescription in New York compared with California (Table 2 and eTable 2 in Supplement 1). Section 3331 was also associated with decreased MMEs per prescription in New York compared with California in the 8 to 30–day period after TJR (−54.89 MMEs; 95% CI, −77.34 to −32.44 MMEs; *P* < .001). We did not identify significant associations between Section 3331 and other MME end points.

**Table 2.** Adjusted Means From Multivariable Hierarchical Regression Models Examining the Association of Section 3331 With MME in the Post-TJR Period

Time since discharge	Estimate, mean (95% CI)^a^						
	California			New York			Difference in differences^b^
	Before implementation	After implementation	Difference	Before implementation	After implementation	Difference	
**Total MMEs**							
≤7 d (n = 57 098)	743.37 (713.20-773.54)	608.29 (578.51-638.08)	−135.08 (−146.62 to −123.53)^c^	651.88 (609.04-694.73)	473.89 (431.93-515.84)	−178.00 (−191.98 to −164.02)^c^	−42.92^c^ (−61.04 to −24.80)
8-30 d (n = 36 462)	869.33 (838.83-899.83)	716.82 (687.81-745.84)	−152.50 (−173.80 to −131.21)^c^	781.70 (731.05-832.36)	589.65 (543.28-636.03)	−192.05 (−225.47 to −158.63)^c^	−39.54 (−79.13 to 0.04)
31-90 d (n = 24 713)	1313.06 (1248.41-1377.72)	1067.08 (1007.55-1126.61)	−245.98 (−311.70 to −180.26)^c^	1156.69 (1037.57-1275.81)	942.75 (839.69-1045.82)	−213.94 (−323.21 to −104.66)^c^	32.04 (−95.30 to 159.39)
**MMEs per prescription**							
≤7 d (n = 57 098)	599.20 (573.83-624.56)	558.02 (532.82-583.21)	−41.18 (−48.45 to −33.91)^c^	550.93 (514.76-587.10)	449.42 (413.67-485.17)	−101.51 (−110.31 to −92.70)^c^	−60.33^c^ (−71.74 to −48.91)
8-30 d (n = 36 462)	617.15 (593.70-640.59)	525.05 (502.17-547.92)	−92.10 (−104.16 to −80.05)^c^	588.70 (550.69-626.71)	441.71 (405.55-477.87)	−146.99 (−165.94 to −128.04)^c^	−54.89^c^ (−77.34 to −32.44)
31-90 d (n = 24 713)	655.78 (630.69-680.87)	560.58 (536.62-584.53)	−95.20 (−115.14 to −75.27)^c^	622.18 (577.58-666.78)	498.16 (457.50-538.81)	−124.02 (−157.24 to −90.80)^c^	−28.82 (−67.53 to 9.89)
**MMEs per day**							
≤7 d (n = 57 098)	64.08 (62.14-66.03)	58.29 (56.36-60.22)	−5.80 (−6.39 to −5.21)^c^	54.13 (51.36-56.90)	48.76 (46.03-51.49)	−5.37 (−6.09 to −4.66)^c^	0.42 (−0.50 to 1.35)
8-30 d (n = 36 462)	58.17 (56.64-59.70)	51.20 (49.71-52.70)	−6.97 (−7.70 to −6.24)^c^	48.31 (45.85-50.78)	41.64 (39.28-43.99)	−6.68 (−7.83 to −5.53)^c^	0.29 (−1.07 to 1.65)
31-90 d (n = 24 713)	48.26 (46.94-49.57)	42.17 (40.90-43.45)	−6.08 (−6.96 to −5.21)^c^	41.46 (39.17-43.74)	35.83 (33.69-37.96)	−5.63 (−7.09 to −4.17)^c^	0.45 (−1.25 to 2.15)

Abbreviations: MME, morphine milligram equivalents; TJR, total joint replacement.

^a^ Estimates are derived from regression models presented in eTable 2 in Supplement 1.

^b^ Calculated as the difference in California subtracted from the difference in New York.

^c^ *P* < .001.

#### Opioid Fills

Change in likelihood of filling at least 1 opioid prescription in the 7-day post-TJR period was −7.76 percentage points (95% CI, −8.57 to −6.96 percentage points; *P* < .001) in California and −5.27 percentage points (95% CI, −6.40 to −4.13 percentage points; *P* < .001) in New York after Section 3331 was implemented compared with before (Table 3 and eTable 3 in Supplement 1). However, because the decrease in New York was smaller than in California, Section 3331 was associated with an increase in likelihood of 2.49 percentage points (95% CI, 1.12-3.88 percentage points; *P* < .001) (Table 3) in New York compared with California. The law was also associated with increased likelihood of at least 1 opioid prescription fill during the 31 to 90–day period in New York compared with California.

**Table 3.** Adjusted Estimates From Multivariable Hierarchical Regression Models Examining the Association of Section 3331 With Opioid Fills in the Post-TJR Period

Time since discharge	Estimate, mean (95% CI)^a^						
	California			New York			Difference in differences^b^
	Before implementation	After implementation	Difference	Before implementation	After implementation	Difference	
**Likelihood of at least 1 opioid fill, %** ^c^							
≤7 d (n = 92 058)	63.04 (60.06-66.03)	55.28 (52.17-58.40)	−7.76 (−8.57 to −6.96)^d^	72.49 (68.47-76.50)	67.22 (62.91-71.53)	−5.27 (−6.40 to −4.13)^d^	2.49 (1.12 to 3.88)^d^
8-30 d (n = 92 058)	45.02 (43.67-46.38)	41.65 (40.37-42.93)	−3.37 (−4.16 to −2.59)^d^	37.06 (35.07-39.05)	33.52 (31.73-35.31)	−3.54 (−4.60 to −2.47)^d^	−0.17 (−1.48 to 1.15)
31-90 d (n = 92 058)	33.10 (32.06-34.15)	26.95 (26.08-27.83)	−6.15 (−6.87 to −5.42)^d^	25.35 (23.91-26.78)	20.79 (19.65-21.93)	−4.56 (−5.50 to −3.62)^d^	1.59 (0.41 to 2.78)^d^
**Opioid fills, No.**							
≤7 d (n = 57 098)	1.25 (1.23-1.28)	1.23 (1.20-1.25)	−0.02 (−0.03 to −0.01)^d^	1.20 (1.16-1.23)	1.20 (1.16-1.23)	0.00 (−0.01 to 0.01)	0.02 (0.01 to 0.04)^e^
8-30 d (n = 36 462)	1.39 (1.37-1.41)	1.37 (1.35-1.39)	−0.02 (−0.04 to 0.00)^f^	1.33 (1.29-1.36)	1.34 (1.31-1.38)	0.02 (−0.01 to 0.05)	0.04 (0.01 to 0.07)^f^
31-90 d (n = 24 713)	1.77 (1.74-1.79)	1.68 (1.65-1.71)	−0.09 (−0.12 to −0.06)^d^	1.60 (1.55-1.65)	1.66 (1.62-1.71)	0.06 (0.02 to 0.11)^f^	0.15 (0.09 to 0.21)^d^

Abbreviation: TJR, total joint replacement.

^a^ The estimates are derived from regression models presented in eTable 3 in Supplement 1.

^b^ Calculated as the difference in California subtracted from the difference in New York.

^c^ Differences are presented as percentage points.

^d^ *P* < .001.

^e^ *P* < .01.

^f^ *P* < .05.

Compared with before Section 3331 implementation, the number of opioid fills in the 7-day post-TJR period decreased in California and remained unchanged in New York, resulting in a small Section 3331–associated increase (estimate, 0.02; 95% CI, 0.01-0.04; *P* = .01) in the number of fills in New York compared with California. The law was also associated with an increased number of opioid fills in New York compared with California in the periods from 8 to 30 days and 31 to 90 days after TJR.

#### Days’ Supply of Opioids

Section 3331 was associated with a change of −24.77 percentage points (95% CI, −26.74 to −22.80 percentage points; *P* < .001) in the likelihood of an opioid prescription being longer than 7 days in the 7-day post-TJR period in New York compared with California (Table 4 and eTable 4 in Supplement 1). Total days’ supply in the 7-day post-TJR period remained unchanged in California and changed by −1.58 days (95% CI, −1.79 to −1.37 days; *P* < .001) in New York (Table 4), resulting in a Section 3331–associated decline in the total days’ supply in New York compared with California (estimate, −1.55 days; 95% CI, −1.82 to −1.28 days; *P* < .001). The law was also associated with a decline in total days’ supply for prescriptions filled in the 8 to 30–day post-TJR period in New York compared with California. Section 3331 was associated with a decrease in the estimated days’ supply per prescription in all 3 periods in New York compared with California (≤7 days post-TJR: −1.58 [95% CI, −1.76 to −1.40] days’ supply [*P* < .001]; 8-30 days post-TJR: −1.35 [95% CI, −1.68 to −1.02] days’ supply [*P* < .001]; 31-90 days post-TJR: −0.81 [95% CI, −1.41 to −0.20] days’ supply [*P* = .009]).

**Table 4.** Adjusted Estimates From Multivariable Hierarchical Regression Models Examining the Association of Section 3331 With Opioid Days’ Supply in the Post-TJR Period

Time since discharge	Estimate, mean (95% CI)^a^						
	California			New York			Difference in differences^b^
	Before implementation	After implementation	Difference	Before implementation	After implementation	Difference	
**Likelihood of opioid prescription being >7 d, %** ^c^							
≤7 d (n = 57 098)	67.04 (64.00-70.07)	55.62 (52.28-58.96)	−11.42 (−12.51 to −10.32)^d^	75.82 (72.16-79.48)	39.64 (34.93-44.34)	−36.19 (−37.99 to −34.38)^d^	−24.77 (−26.74 to −22.80)^d^
**Total days’ supply**							
7 d (n = 57 098)	13.05 (12.63-13.48)	13.02 (12.60-13.44)	−0.03 (−0.21 to 0.14)	13.07 (12.46-13.67)	11.48 (10.89-12.08)	−1.58 (−1.79 to −1.37)^d^	−1.55 (−1.82 to −1.28)^d^
8-30 d (n = 36 462)	16.43 (15.98-16.88)	15.30 (14.87-15.74)	−1.13 (−1.41 to −0.85)^d^	17.15 (16.41-17.89)	14.74 (14.05-15.43)	−2.42 (−2.86 to −1.97)^d^	−1.29 (−1.81 to −0.76)^d^
31-90 d (n = 24 713)	28.17 (27.46-28.89)	26.00 (25.33-26.67)	−2.18 (−2.83 to −1.52)^d^	27.27 (25.98-28.57)	25.36 (24.21-26.51)	−1.91 (−3.00 to −0.83)^d^	0.27 (−1.00 to 1.53)
**Days’ supply per prescription**							
≤7 d (n = 57 098)	10.42 (10.08-10.75)	10.78 (10.45-11.11)	0.36 (0.25 to 0.47)^d^	10.86 (10.39-11.34)	9.64 (9.17-10.11)	−1.22 (−1.36 to −1.08)^d^	−1.58 (−1.76 to −1.40)^d^
8-30 d (n = 36 462)	12.07 (11.67-12.47)	11.47 (11.08-11.86)	−0.60 (−0.78 to −0.42)^d^	13.36 (12.72-14.00)	11.40 (10.79-12.02)	−1.95 (−2.23 to −1.67)^d^	−1.35 (−1.68 to −1.02)^d^
31-90 d (n = 24 713)	15.87 (15.43-16.32)	15.16 (14.73-15.59)	−0.71 (−1.02 to −0.40)^d^	16.38 (15.60-17.16)	14.86 (14.14-15.59)	−1.52 (−2.04 to −1.00)^d^	−0.81 (−1.41 to −0.20)^e^

Abbreviation: TJR, total joint replacement.

^a^ These estimates are derived from regression models presented in eTable 4 in Supplement 1.

^b^ Calculated as the difference in California subtracted from the difference in New York.

^c^ Differences are presented as percentage points.

^d^ *P* < .001.

^e^ *P* < .01.

### Sensitivity and Secondary Analyses

Inferences from sensitivity and secondary analyses (eTables 5 [variable preimplementation phase], 6 [outpatient total knee replacement inclusion], 7 [total hip replacement cohort], 8 [total knee replacement cohort], 9 [opioid-naive patients only], and 12 and 13 [cumulative 90- and 30-day post-TJR periods, respectively] in Supplement 1) were consistent with the main analysis. In the analysis by race and ethnicity, likelihood of an opioid fill longer than 7 days (triple-differences estimate, −16.21 percentage points; 95% CI, −30.32 to −2.11 percentage points; *P* = .02) and days’ supply per prescription (triple-differences estimate, −1.81 days; 95% CI, −3.50 to −0.12 days; *P* = .04) declined for Hispanic patients compared with White patients after Section 3331 implementation (eTable 10 in Supplement 1). The association of Section 3331 with end points did not vary across dual-eligible and non–dual-eligible patients (eTable 11 in Supplement 1).

## Discussion

In our evaluation of a New York law capping opioid prescribing following TJR for Medicare beneficiaries, we found that the legislation was associated with a decrease in the total quantity of opioids filled in the immediate 7 days following TJRs in New York compared with California. This reduction likely was attributable to decreases in the quantity of opioids filled per prescription and the duration of opioid prescriptions in New York compared with California. Section 3331 was also associated with a smaller decline in the likelihood of an opioid fill in New York compared with California, and the number of fills marginally increased. The legislation had variable associations with end points in the longer postoperative period. We found that the law was associated with declines in the likelihood of opioid prescriptions longer than 7 days and the days’ supply per prescription for Hispanic patients compared with White patients. Overall, our findings suggest that Section 3331 may have achieved its intended objective of reducing opioid prescribing for acute pain in the short-term 7-day post-TJR period. To our knowledge, our study is the first comprehensive evaluation of the New York SOCL for TJRs and provides a model for examining other SOCLs.

SOCLs such as Section 3331 are intended to limit opioid use by reducing the amounts of opioids prescribed (and subsequently dispensed and filled), thereby reducing exposure of patients to prolonged durations of opioids, limiting the risk of addiction to these drugs, and reducing the quantity of leftover opioids that may be diverted for misuse in the community. The net decline in the total quantity, or MMEs, of opioids prescribed that we found in our study is consistent with the intended effects of the legislation and the results of other single-center studies and 1 national study examining the association of cap laws with opioid prescribing.^15,20,21,22,23,24,25,26,27^ These findings suggest that New York Section 3331 and legislation in other states have been effective in reducing the amount of opioids prescribed and consequently left unused, at least in the immediate post-TJR period. However, much work remains to restrict opioid prescribing in the later post-TJR period, suggesting there is an opportunity to refine Section 3331 to reduce perioperative prescribing. For Section 3331, whether the law can be strengthened by including duration limits for subsequent prescriptions (and not only the first prescription), as in Michigan, or whether quantity limits need to be introduced along with duration limits, as in Ohio, are both areas for future research.^42^

The increase in the percentage of patients exposed to opioids and the number of opioid fills following the implementation of legislation has been previously noted.^15,20,25^ These changes may have intentionally resulted from adherence to national guidelines that advocate opioid prescriptions of variable strengths to support the tapering of these drugs.^15,43^ On the other hand, these changes may have resulted from patients returning more often for additional fills. These findings reflect the flexibility in opioid prescribing practices, which can be further leveraged to achieve additional reductions. The findings could also be explained by prescribing uncertainty driven by the complexity of the law and the lack of clarity in understanding its nuances.^44^

Our finding of the presence of prescriptions longer than 7 days in New York even after the implementation of Section 3331 merits further investigation. An important explanation for this finding may be the inadequate enforcement of the law across New York. Per the law, prescribers and not pharmacists are responsible for compliance.^45^ However, several barriers, which include the complexity of the law, lack of prescriber understanding of the law, exemptions to the law, and information technology infrastructure, may result in inadequate enforcement by physicians.^44^

The differential reduction in opioid prescriptions longer than 7 days and the days’ supply per prescription for Hispanic patients compared with White patients may have result from relatively higher rates of these metrics for Hispanic patients before the implementation of the law, thereby providing an opportunity for a greater reduction after the passage of the law. However, whether these changes help in optimizing the availability of opioids for Hispanic patients or whether they create barriers to managing their pain are open questions and need detailed analyses.

Our findings have several important policy and practice implications. States with similar SOCLs may wish to consider refining their laws to achieve their intended end points. Currently, there is considerable heterogeneity in SOCL design, with 13 unique SOCL phenotypes resulting from the type of opioid restriction (restriction of duration, quantity, or both), exceptions for surgical pain and professional judgment, and inclusion of only initial or all subsequent opioid prescriptions for acute pain. Second, 11 states and the District of Columbia have never implemented SOCLs. Among these, the District of Columbia and states such as New Mexico have opioid-related mortality rates that are above the national average.^46^ In addition, our study provides evidence to help patients calibrate their expectations of pain relief, for physicians to discuss options for post-TJR analgesia consistent with Section 3331 restrictions, and to identify targets for prescriber education and awareness.

### Limitations

Our study has limitations. First, our study focused on fee-for-service Medicare patients. Hence, our findings have limited generalizability to 39% of Medicare beneficiaries enrolled in Medicare Advantage in 2019.^47^ Second, we focused on opioid prescription end points. Whether Section 3331–associated changes in opioid prescribing are associated with meaningful changes in the pain needs of patients, morbidity, or mortality is an area for future research. Third, we were unable to reliably examine prescribing for nonopioid analgesics, as many of them are over-the-counter drugs, and hence, their claims are not included in the Part D data. Fourth, although patients in New York and California differed in important ways, the use of the DID approach allowed us to estimate the independent association of Section 3331 with opioid prescribing based on the assumption that in the absence of the law, the end points in New York and California in the post–Section 3331 period would have trended similarly to the pre-Section 3331 period. Finally, there may be several other contemporaneous events, both published and unpublished, that may have influenced opioid use. These include hospital-level practice changes and growing evidence about the effectiveness of nonopioid analgesics. While it is challenging to account for every such event implemented at the state or hospital level, our use of the DID approach and hospital-level random effects, accounting for diverging linear trends where appropriate, and a range of sensitivity analyses strengthen our key findings.

## Conclusions

In this cohort study, New York Section 3331 was associated with reductions in opioids filled during the 7-day post-TJR period in New York compared with California. The legislation was not associated with meaningful reductions in MMEs filled in the later post-TJR periods. Whether the current design of Section 3331 suffices to restrict opioid use for TJRs, one of the highest-priority procedures, or whether refinements are needed to achieve further reductions are open questions. Our findings provide a rigorous foundation to test these important clinical and policy questions.

## Notes

Supplement 1. **eMethods 1.** Medicare Severity Diagnosis–Related Groups and ICD-9 and ICD-10 Procedure Codes Used for Identifying the Cohort **eMethods 2.** Cohort Determination Criteria for New York and California **eMethods 3.** Covariates Controlled for in Regression Models **eMethods 4.** Model Specifications **eMethods 5.** Sensitivity and Secondary Analyses **eTable 1.** Hospital Characteristics in the Pre–Section 3331 Period **eTable 2.** Estimates From Multivariable Hierarchical Linear Regression Models Examining the Association of Section 3331 MME in the Post-TJR Period **eTable 3.** Estimates From Multivariable Hierarchical Regression Models Examining the Association of Section 3331 With Opioid Fills in the Post-TJR Period **eTable 4.** Estimates From Multivariable Hierarchical Regression Models Examining the Association of Section 3331 With Opioid Days’ Supply in the Post-TJR Period **eTable 5.** Adjusted Estimates for Association of Section 3331 With End Points From Discharge to 7 Days Post-TJR for Pre–Section 3331 Phase Ending in July 2016 **eTable 6.** Adjusted Estimates for Association of Section 3331 With End Points From Discharge to 7 Days Post-TJR for Outpatient Total Knee Replacements, 2018-2019 **eTable 7.** Adjusted Estimates for Association of Section 3331 With End Points From Discharge to 7 Days Post-TJR for Total Hip Replacements **eTable 8.** Adjusted Estimates for Association of Section 3331 With End Points from Discharge to 7 Days Post-TJR for Total Knee Replacements **eTable 9.** Adjusted Estimates for Association of Section 3331 With End Points From Discharge to 7 Days Post-TJR Among Opioid-Naive Patients Only **eTable 10.** Adjusted Estimates for Association of Section 3331 With End Points From Discharge to 7 Days Post-TJR by Race and Ethnicity **eTable 11.** Adjusted Estimates for Association of Section 3331 With End Points From Discharge to 7 Days Post-TJR for Dual-Eligible and Non–Dual-Eligible Patients **eTable 12.** Adjusted Estimates for Association of Section 3331 With End Points in the Cumulative 90-Day Post-TJR Period **eTable 13.** Adjusted Estimates for Association of Section 3331 With End Points in the Cumulative 30-Day Post-TJR Period
eReferences.

Supplement 2.
Data Sharing Statement
