# Supplementary material for: Opioid Cap Laws and Opioid Prescriptions After Total Joint Replacements in Older Adults
Source: JAMA Netw Open. 2025 Apr 9;8(4):e254448. doi: 10.1001/jamanetworkopen.2025.4448 (PMC11983234; doi:10.1001/jamanetworkopen.2025.4448)
Supplement: Supplement 1. — eMethods 1. Medicare Severity Diagnosis–Related Groups and ICD-9 and ICD-10 Procedure Codes Used for Identifying the Cohort eMethods 2. Cohort Determination Criteria for New York and California eMethods 3. Covariates Controlled for in Regression Models eMethods 4. Model Specifications eMethods 5. Sensitivity and Secondary Analyses eTable 1. Hospital Characteristics in the Pre–Section 3331 Period eTable 2. Estimates From Multivariable Hierarchical Linear Regression Models Examining the Association of Section 3331 MME in the Post-TJR Period eTable 3. Estimates From Multivariable Hierarchical Regression Models Examining the Association of Section 3331 With Opioid Fills in the Post-TJR Period eTable 4. Estimates From Multivariable Hierarchical Regression Models Examining the Association of Section 3331 With Opioid Days’ Supply in the Post-TJR Period eTable 5. Adjusted Estimates for Association of Section 3331 With End Points From Discharge to 7 Days Post-TJR for Pre–Section 3331 Phase Ending in July 2016 eTable 6. Adjusted Estimates for Association of Section 3331 With End Points From Discharge to 7 Days Post-TJR for Outpatient Total Knee Replacements, 2018-2019 eTable 7. Adjusted Estimates for Association of Section 3331 With End Points From Discharge to 7 Days Post-TJR for Total Hip Replacements eTable 8. Adjusted Estimates for Association of Section 3331 With End Points from Discharge to 7 Days Post-TJR for Total Knee Replacements eTable 9. Adjusted Estimates for Association of Section 3331 With End Points From Discharge to 7 Days Post-TJR Among Opioid-Naive Patients Only eTable 10. Adjusted Estimates for Association of Section 3331 With End Points From Discharge to 7 Days Post-TJR by Race and Ethnicity eTable 11. Adjusted Estimates for Association of Section 3331 With End Points From Discharge to 7 Days Post-TJR for Dual-Eligible and Non–Dual-Eligible Patients eTable 12. Adjusted Estimates for Association of Section 3331 With End Points in the Cumulative 90-Day Post [file jamanetwopen-e254448-s001.pdf]

Supplementary Online Content

Thirukumaran CP, Schloemann DT, Doshi JA, Fiscella KA, Ricciardi BF, Rosenthal MB. Opioid cap laws and opioid prescriptions after total joint replacements in older adults. *JAMA Netw Open*. 2025;8(4):e254448. doi:10.1001/jamanetworkopen.2025.4448

eMethods 1. Medicare Severity Diagnosis–Related Groups and *ICD-9* and *ICD-10* Procedure Codes Used for Identifying the Cohort
eMethods 2. Cohort Determination Criteria for New York and California
eMethods 3. Covariates Controlled for in Regression Models
eMethods 4. Model Specifications
eMethods 5. Sensitivity and Secondary Analyses
eTable 1. Hospital Characteristics in the Pre–Section 3331 Period
eTable 2. Estimates From Multivariable Hierarchical Linear Regression Models Examining the Association of Section 3331 MME in the Post-TJR Period
eTable 3. Estimates From Multivariable Hierarchical Regression Models Examining the Association of Section 3331 With Opioid Fills in the Post-TJR Period
eTable 4. Estimates From Multivariable Hierarchical Regression Models Examining the Association of Section 3331 With Opioid Days' Supply in the Post-TJR Period
eTable 5. Adjusted Estimates for Association of Section 3331 With End Points From Discharge to 7 Days Post-TJR for Pre–Section 3331 Phase Ending in July 2016
eTable 6. Adjusted Estimates for Association of Section 3331 With End Points From Discharge to 7 Days Post-TJR for Outpatient Total Knee Replacements, 2018-2019
eTable 7. Adjusted Estimates for Association of Section 3331 With End Points From Discharge to 7 Days Post-TJR for Total Hip Replacements
eTable 8. Adjusted Estimates for Association of Section 3331 With End Points from Discharge to 7 Days Post-TJR for Total Knee Replacements
eTable 9. Adjusted Estimates for Association of Section 3331 With End Points From Discharge to 7 Days Post-TJR Among Opioid-Naive Patients Only
eTable 10. Adjusted Estimates for Association of Section 3331 With End Points From Discharge to 7 Days Post-TJR by Race and Ethnicity
eTable 11. Adjusted Estimates for Association of Section 3331 With End Points From Discharge to 7 Days Post-TJR for Dual-Eligible and Non–Dual-Eligible Patients
eTable 12. Adjusted Estimates for Association of Section 3331 With End Points in the Cumulative 90-Day Post-TJR Period
eTable 13. Adjusted Estimates for Association of Section 3331 With End Points in the Cumulative 30-Day Post-TJR Period
eReferences

This supplementary material has been provided by the authors to give readers additional information about their work.

© 2025 Thirukumaran CP et al. *JAMA Network Open*

eMethods 1: Medicare Severity Diagnosis Related Groups and International Classification of Diseases (Ninth and Tenth Revision) Procedure Codes used for identifying the cohort

Code	Code Description
MS-DRG	
469	Major joint replacement or reattachment of lower extremity with major complication or comorbidity
470	Major joint replacement or reattachment of lower extremity without major complication or comorbidity
ICD-9-PCS	
8151	Total hip replacement
8154	Total knee replacement
ICD-10-PCS	
0SR9019	Replacement of Right Hip Joint with Metal Synthetic Substitute, Cemented, Open Approach
0SR901A	Replacement of Right Hip Joint with Metal Synthetic Substitute, Uncemented, Open Approach
0SR901Z	Replacement of Right Hip Joint with Metal Synthetic Substitute, Open Approach
0SR9029	Replacement of Right Hip Joint with Metal on Polyethylene Synthetic Substitute, Cemented, Open Approach
0SR902A	Replacement of Right Hip Joint with Metal on Polyethylene Synthetic Substitute, Uncemented, Open Approach
0SR902Z	Replacement of Right Hip Joint with Metal on Polyethylene Synthetic Substitute, Open Approach
0SR9039	Replacement of Right Hip Joint with Ceramic Synthetic Substitute, Cemented, Open Approach
0SR903A	Replacement of Right Hip Joint with Ceramic Synthetic Substitute, Uncemented, Open Approach
0SR903Z	Replacement of Right Hip Joint with Ceramic Synthetic Substitute, Open Approach
0SR9049	Replacement of Right Hip Joint with Ceramic on Polyethylene Synthetic Substitute, Cemented, Open Approach
0SR904A	Replacement of Right Hip Joint with Ceramic on Polyethylene Synthetic Substitute, Uncemented, Open Approach
0SR904Z	Replacement of Right Hip Joint with Ceramic on Polyethylene Synthetic Substitute, Open Approach
0SR9069	Replacement of Right Hip Joint with Oxidized Zirconium on Polyethylene Synthetic Substitute, Cemented, Open Approach
0SR906A	Replacement of Right Hip Joint with Oxidized Zirconium on Polyethylene Synthetic Substitute, Uncemented, Open Approach
0SR906Z	Replacement of Right Hip Joint with Oxidized Zirconium on Polyethylene Synthetic Substitute, Open Approach
0SR90J9	Replacement of Right Hip Joint with Synthetic Substitute, Cemented, Open Approach
0SR90JA	Replacement of Right Hip Joint with Synthetic Substitute, Uncemented, Open Approach
0SR90JZ	Replacement of Right Hip Joint with Synthetic Substitute, Open Approach
0SRB019	Replacement of Left Hip Joint with Metal Synthetic Substitute, Cemented, Open Approach
0SRB01A	Replacement of Left Hip Joint with Metal Synthetic Substitute, Uncemented, Open Approach
0SRB01Z	Replacement of Left Hip Joint with Metal Synthetic Substitute, Open Approach
0SRB029	Replacement of Left Hip Joint with Metal on Polyethylene Synthetic Substitute, Cemented, Open Approach
0SRB02A	Replacement of Left Hip Joint with Metal on Polyethylene Synthetic Substitute, Uncemented, Open Approach

© 2025 Thirukumaran CP et al. *JAMA Network Open*

Code	Code Description
0SRB02Z	Replacement of Left Hip Joint with Metal on Polyethylene Synthetic Substitute, Open Approach
0SRB039	Replacement of Left Hip Joint with Ceramic Synthetic Substitute, Cemented, Open Approach
0SRB03A	Replacement of Left Hip Joint with Ceramic Synthetic Substitute, Uncemented, Open Approach
0SRB03Z	Replacement of Left Hip Joint with Ceramic Synthetic Substitute, Open Approach
0SRB049	Replacement of Left Hip Joint with Ceramic on Polyethylene Synthetic Substitute, Cemented, Open Approach
0SRB04A	Replacement of Left Hip Joint with Ceramic on Polyethylene Synthetic Substitute, Uncemented, Open Approach
0SRB04Z	Replacement of Left Hip Joint with Ceramic on Polyethylene Synthetic Substitute, Open Approach
0SRB069	Replacement of Left Hip Joint with Oxidized Zirconium on Polyethylene Synthetic Substitute, Cemented, Open Approach
0SRB06A	Replacement of Left Hip Joint with Oxidized Zirconium on Polyethylene Synthetic Substitute, Uncemented, Open Approach
0SRB06Z	Replacement of Left Hip Joint with Oxidized Zirconium on Polyethylene Synthetic Substitute, Open Approach
0SRB0J9	Replacement of Left Hip Joint with Synthetic Substitute, Cemented, Open Approach
0SRB0JA	Replacement of Left Hip Joint with Synthetic Substitute, Uncemented, Open Approach
0SRB0JZ	Replacement of Left Hip Joint with Synthetic Substitute, Open Approach
0SRC069	Replacement of Right Knee Joint with Oxidized Zirconium on Polyethylene Synthetic Substitute, Cemented, Open Approach
0SRC06A	Replacement of Right Knee Joint with Oxidized Zirconium on Polyethylene Synthetic Substitute, Uncemented, Open Approach
0SRC06Z	Replacement of Right Knee Joint with Oxidized Zirconium on Polyethylene Synthetic Substitute, Open Approach
0SRC0J9	Replacement of Right Knee Joint with Synthetic Substitute, Cemented, Open Approach
0SRC0JA	Replacement of Right Knee Joint with Synthetic Substitute, Uncemented, Open Approach
0SRC0JZ	Replacement of Right Knee Joint with Synthetic Substitute, Open Approach
0SRD069	Replacement of Left Knee Joint with Oxidized Zirconium on Polyethylene Synthetic Substitute, Cemented, Open Approach
0SRD06A	Replacement of Left Knee Joint with Oxidized Zirconium on Polyethylene Synthetic Substitute, Uncemented, Open Approach
0SRD06Z	Replacement of Left Knee Joint with Oxidized Zirconium on Polyethylene Synthetic Substitute, Open Approach
0SRD0J9	Replacement of Left Knee Joint with Synthetic Substitute, Cemented, Open Approach
0SRD0JA	Replacement of Left Knee Joint with Synthetic Substitute, Uncemented, Open Approach
0SRD0JZ	Replacement of Left Knee Joint with Synthetic Substitute, Open Approach

eMethods 2: Cohort determination criteria for New York and California

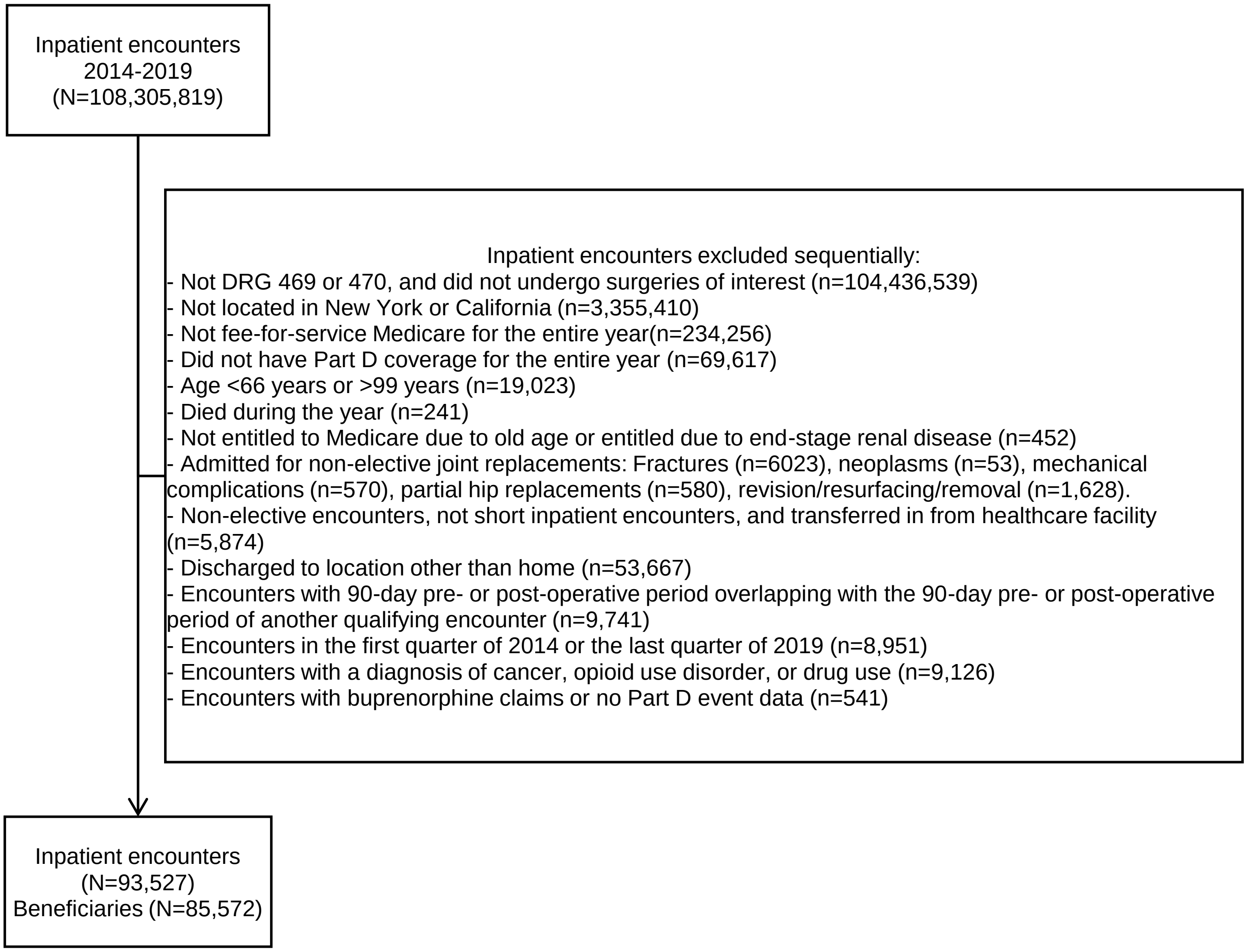

© 2025 Thirukumaran CP et al. *JAMA Network Open*

eMethods 3: Covariates controlled for in regression models

In multivariable analysis, the regression models controlled for patient-level covariates, which included continuous indicators for age and categorical indicators for sex (male or female), race/ethnicity as reported in the Social Security Administration records[1] and classified in the Medicare Master Beneficiary Summary file (Asian, Black, Hispanic, North American Native, Other, Unknown, and White), dual eligibility for Medicare and Medicaid (a marker for socioeconomic risk), type of surgery (total hip or knee replacement), discharge destination (home with self-care or home with home healthcare), three or more opioid or benzodiazepine fills in the 90 days before admission, Elixhauser's comorbidities, mental health conditions (anxiety, bipolar disorder, depression, personality disorder, post-traumatic stress disorder, psychosis, schizophrenia and other psychotic disorder), and alcohol and tobacco use disorder.

Hospital-level controls included categorical indicators for ownership (private for-profit, private not-for-profit, and government), number of beds (small [<200], medium [>=200 and <400], and large [>=400]), and quartiles of annual hospital TJR volume; binary indicators for urban location and medical school affiliation; and continuous indicators for disproportionate patient percentage share index (a marker for the clinical and socially complex patient volume) and transfer-adjusted case mix of the hospital.

© 2025 Thirukumaran CP et al. *JAMA Network Open*

eMethods 4: Model specifications

<u>Test for parallel trends assumption for the difference-in-differences models:</u>

To assess whether the trends in the endpoints were parallel in NY and CA in the period before the Section 3331 was implemented, we estimated the following models using the pre-Section 3331 data.

Eq 1: $f\left[E\left(Y_p\right)\right] = \beta_0 + \beta_1 \times State_s + \beta_2 \times YrQt_t + \beta_3 \times State_s \times YrQt_t + \beta_4 \times Patient_p + \beta_5 \times Hospital_h + e_h$

Y_p : Endpoint of interest

$f(.)$: Link function

$State_s$: Binary indicator for treatment (NY) or control (CA) state

$YrQt_t$: Year-quarter indicator modeled as a continuous variable

$Patient_p$: Vector of patient covariates

$Hospital_h$: Vector of hospital covariates

e_h: Hospital clustering modeled using hospital-level random effects

We used the Wald test to test for the significance of β_3. A p-value <0.05 represented a violation of the parallel trends assumption.

<u>Results of the parallel trends tests for triple differences models</u>

The parallel trends assumption was violated for the MME/script, total days supply, and the days supply per script endpoints in the 7-day period. The assumption was not violated for endpoints in the 8-to-30- or 31-to-90-day period in the main analysis.

© 2025 Thirukumaran CP et al. *JAMA Network Open*

Model specification for difference-in-differences models without violation of parallel trends assumption

Eq 2: $f\left[E\left(Y_p\right)\right] = \beta_0 + \beta_1 \times State_s + \beta_2 \times Policy_t + \beta_3 \times State_s \times Policy_t + \beta_4 \times Patient_p + \beta_5 \times Hospital_h + e_h$

$Policy_t$: Binary indicator for whether the observation was from the period before or after the implementation of Section 3331 in NY.

Model estimation for difference-in-difference models where the parallel trends assumption was violated

To account for the differential trends in the pre-Section 3331 period, we used the approach by Bilinski and Hatfield[2] and Nakamoto et al.[3] Specifically, for the endpoints where the assumption was violated,

- We obtained the interaction coefficient from equation 1 – this coefficient represents the differential linear trend in the endpoint between NY and CA in the pre-Section 3331 period.
- Next, to project this trend in the post-Section 3331 period, we multiplied this coefficient by 10 (this is the number of quarters between the midpoints of the pre- and post-Section 3331 periods), subtracted this value from the endpoint values in the post-Section 3331 period (trend-adjusted endpoint), and finally estimated equation 2 using this trend-adjusted endpoint.

© 2025 Thirukumaran CP et al. *JAMA Network Open*

eMethods 5: Sensitivity and secondary analysis

To test the robustness of our findings and how Section 3331 may have influenced opioid prescribing for different subgroups, we conducted several sensitivity and secondary analysis. Unless otherwise stated, these models were estimated for endpoints in the 7-day post-TJR period, which coincides with the duration limit of Section 3331. *First,* because prescribers may need additional time to streamline their systems, we redefined the pre-Section 3331 phase to end in July 2016 (instead of June 2016). *Second,* total knee replacements were removed from Medicare's inpatient-only list in 2018 and started to be performed in hospital outpatient departments.[4] Hence we used Medicare's 2018-2019 outpatient and carrier files to identify total knee replacements performed in hospital outpatient departments and included them in the regression models along with an indicator for whether a surgery was performed on an inpatient or outpatient basis. *Third,* we re-estimated the regression models separately in the total hip and knee replacement cohorts. *Fourth,* we re-estimated the models among patients who had no opioid fills in the 90 days before admission to determine whether Section 3331 may have differential effects among opioid naïve patients. *Fifth,* we estimated whether the association of Section 3331 with the endpoints varied for patients belonging to various race/ethnicity groups. For this, we limited the cohort to Black, Hispanic, and White patients as determined by the race/ethnicity information included in the MBSF data. This led to the exclusion of 4,379 patients whose race/ethnicity was reported as either Other or Unknown and 2,107 patients identifying as Asian or North American Native due to their smaller sample size. These regression models used a triple differences approach which included an interaction of the phase (before/after), state (NY/CA), and race/ethnicity variables along with their main effects and lower-order interactions. *Sixth,* using the triple differences approach, we estimated whether the association varied for patients from higher or lower socioeconomic groups as determined by their dual-eligibility for Medicare and Medicaid. *Finally,* we determined the association for the cumulative 30- and 90-day post-TJR period to understand the overall long-term trends.

© 2025 Thirukumaran CP et al. *JAMA Network Open*

Note that the estimation of all of these models for the sensitivity and secondary analyses, including testing for the parallel trends assumption, followed the approaches detailed in eMethods 4.

© 2025 Thirukumaran CP et al. *JAMA Network Open*

eTable 1: Hospital characteristics in the pre-Section 3331 period (April 2014 to June 2016)

	**California**	**New York**	**Total**	**p-value**
Hospitals: N	237	119	356	
Ownership: N (%)				<0.001
Private for-profit	51 (21.52)	3 (2.52)	54 (15.17)	
Private not-for-profit	143 (60.34)	107 (89.92)	250 (70.22)	
Government	43 (18.14)	9 (7.56)	52 (14.61)	
Urban: N (%)	230 (97.05)	93 (78.15)	323 (90.73)	<0.001
Number of beds: N (%)				0.003
Small (<200 beds)	118 (49.79)	57 (47.90)	175 (49.16)	
Medium (>=200 and <400 beds)	94 (39.66)	34 (28.57)	128 (35.96)	
Large (>=400 beds)	25 (10.55)	28 (23.53)	53 (14.89)	
Medical school affiliation: N (%)	76 (32.07)	64 (53.78)	140 (39.33)	<0.001
Disproportionate Patient Percentage Share Hospital: Mean (SD)	0.38 (0.23)	0.31 (0.19)	0.35 (0.22)	0.003
Transfer-adjusted casemix index: Mean (SD)	1.64 (0.25)	1.48 (0.25)	1.58 (0.26)	<0.001
Annual TJR volume in quartiles: N (%)				0.37
Quartile 1 (1 to 15)	63 (26.58)	30 (25.21)	93 (26.12)	
Quartile 2 (15 to 42)	54 (22.78)	37 (31.09)	91 (25.56)	
Quartile 3 (42 to 89)	58 (24.47)	27 (22.69)	85 (23.88)	
Quartile 4 (91 to 2017)	62 (26.16)	25 (21.01)	87 (24.44)	

Abbreviations: N: Number, SD: Standard deviation, %: Column percentage, TJR: Total joint replacement

Notes:
P-value from chi-square or Kruskal–Wallis tests comparing the distribution of variables across patients in New York and California.

© 2025 Thirukumaran CP et al. *JAMA Network Open*

eTable 2: Estimates from multivariable hierarchical linear regression models examining the association of Section 3331 with morphine milligram equivalents (MME) in the post-TJR period

	**Total MME**			**MME per script**			**MME per day**		
	7 days	*8-to-30 days*	*31-to-90 days*	*7 days*	*8-to-30 days*	*31-to-90 days*	*7 days*	*8-to-30 days*	*31-to-90 days*
	Estimate [95% CI]	*Estimate [95% CI]*	*Estimate [95% CI]*	*Estimate [95% CI]*	*Estimate [95% CI]*	*Estimate [95% CI]*	*Estimate [95% CI]*	*Estimate [95% CI]*	*Estimate [95% CI]*
**Phase**									
Before	Ref	Ref	Ref	Ref	Ref	Ref	Ref	Ref	Ref
After	-135.08*** [-146.62,-123.53]	-152.50*** [-173.80,-131.21]	-245.98*** [-311.70,-180.26]	-41.18*** [-48.45,-33.91]	-92.10*** [-104.16,-80.05]	-95.20*** [-115.14,-75.27]	-5.80*** [-6.39,-5.21]	-6.97*** [-7.70,-6.24]	-6.08*** [-6.96,-5.21]
**State**									
CA	Ref	Ref	Ref	Ref	Ref	Ref	Ref	Ref	Ref
NY	-91.49*** [-141.43,-41.54]	-87.63** [-147.25,-28.00]	-156.37* [-295.36,-17.38]	-48.27* [-89.71,-6.83]	-28.45 [-72.80,15.91]	-33.60 [-85.17,17.97]	-9.95*** [-13.14,-6.77]	-9.86*** [-12.73,-6.99]	-6.80*** [-9.43,-4.17]
**Phase#State**									
After#NY	-42.92*** [-61.04,-24.80]	-39.54 [-79.13,0.04]	32.04 [-95.30,159.39]	-60.33*** [-71.74,-48.91]	-54.89*** [-77.34,-32.44]	-28.82 [-67.53,9.89]	0.42 [-0.50,1.35]	0.29 [-1.07,1.65]	0.45 [-1.25,2.15]
Age	-9.81*** [-10.62,-9.00]	-14.78*** [-16.49,-13.06]	-28.94*** [-34.36,-23.51]	-6.69*** [-7.20,-6.19]	-7.26*** [-8.23,-6.29]	-8.45*** [-10.09,-6.80]	-0.65*** [-0.69,-0.61]	-0.58*** [-0.63,-0.52]	-0.55*** [-0.63,-0.48]
**Gender**									
Male	Ref	Ref	Ref	Ref	Ref	Ref	Ref	Ref	Ref
Female	-39.46*** [-48.62,-30.31]	-54.32*** [-73.29,-35.35]	-84.04** [-145.40,-22.69]	-34.62*** [-40.38,-28.86]	-46.79*** [-57.51,-36.07]	-50.84*** [-69.41,-32.27]	-4.18*** [-4.65,-3.71]	-4.27*** [-4.92,-3.62]	-3.94*** [-4.76,-3.13]
**Race/ethnicity**									
White	Ref	Ref	Ref	Ref	Ref	Ref	Ref	Ref	Ref
Asian	30.05* [3.70,56.40]	13.88 [-37.34,65.10]	114.20 [-35.77,264.17]	-56.19*** [-75.88,-36.49]	-71.29*** [-106.49,-36.10]	-120.03*** [-179.21,-60.84]	-3.66*** [-5.26,-2.06]	-4.01*** [-6.14,-1.87]	-7.46*** [-10.06,-4.86]
Black	-44.79** [-75.79,-13.78]	-111.33*** [-167.36,-55.30]	-263.67** [-426.03,-101.32]	26.04** [9.44,42.63]	26.92 [-2.12,55.95]	28.25 [-17.37,73.88]	1.12 [-0.22,2.47]	0.44 [-1.32,2.20]	1.00 [-1.01,3.01]
Hispanic	-83.45*** [-114.72,-52.18]	-136.79*** [-198.92,-74.65]	-250.91* [-445.93,-55.88]	-20.86* [-40.40,-1.32]	-57.29*** [-89.07,-25.52]	-101.54*** [-150.92,-52.17]	-2.30** [-3.89,-0.71]	-2.38* [-4.31,-0.46]	-4.11*** [-6.29,-1.94]
North American Native	-65.74 [-168.27,36.79]	-115.08 [-295.25,65.09]	305.36 [-199.54,810.27]	-54.66 [-119.19,9.87]	-40.10 [-142.01,61.81]	101.74 [-51.05,254.53]	-3.66 [-8.90,1.59]	-6.01 [-12.19,0.17]	-1.55 [-8.26,5.16]

© 2025 Thirukumaran CP et al. *JAMA Network Open*

	Total MME			MME per script			MME per day		
	7 days	*8-to-30 days*	*31-to-90 days*	*7 days*	*8-to-30 days*	*31-to-90 days*	*7 days*	*8-to-30 days*	*31-to-90 days*
	Estimate [95% CI]	*Estimate [95% CI]*	*Estimate [95% CI]*	*Estimate [95% CI]*	*Estimate [95% CI]*	*Estimate [95% CI]*	*Estimate [95% CI]*	*Estimate [95% CI]*	*Estimate [95% CI]*
Unknown/other	-30.49** [-50.56,-10.43]	-52.29* [-94.90,-9.68]	-153.39* [-290.87,-15.90]	-18.92** [-31.54,-6.30]	-24.76* [-48.85,-0.68]	-48.97* [-90.59,-7.36]	-1.27* [-2.29,-0.24]	-0.58 [-2.04,0.88]	-2.81** [-4.64,-0.99]
**Dual-eligibility**									
Non-dual-eligible	Ref	Ref	Ref	Ref	Ref	Ref	Ref	Ref	Ref
Dual-eligible	-11.66 [-28.58,5.27]	-6.51 [-37.96,24.93]	83.21 [-9.01,175.43]	-7.27 [-17.93,3.39]	2.93 [-14.91,20.77]	13.67 [-14.42,41.76]	-3.46*** [-4.33,-2.60]	-4.72*** [-5.80,-3.64]	-3.12*** [-4.36,-1.88]
**Surgery**									
Knee replacement	Ref	Ref	Ref	Ref	Ref	Ref	Ref	Ref	Ref
Hip replacement	-118.50*** [-127.46,-109.55]	-157.48*** [-178.01,-136.96]	-148.77*** [-213.20,-84.34]	-67.79*** [-73.43,-62.16]	-49.52*** [-61.13,-37.90]	-14.03 [-33.56,5.50]	-4.95*** [-5.41,-4.49]	-5.12*** [-5.82,-4.41]	-3.30*** [-4.16,-2.44]
**Discharge destination**									
Home	Ref	Ref	Ref	Ref	Ref	Ref	Ref	Ref	Ref
Home with home healthcare	25.30*** [13.72,36.88]	29.78* [6.91,52.66]	75.49* [6.50,144.48]	15.08*** [7.76,22.41]	13.37* [0.17,26.56]	5.17 [-16.53,26.88]	1.92*** [1.33,2.52]	1.32** [0.52,2.12]	0.38 [-0.59,1.35]
**Pre-operative opioid fills**									
Less than 3	Ref	Ref	Ref	Ref	Ref	Ref	Ref	Ref	Ref
3 or more	340.75*** [324.72,356.79]	705.04*** [679.90,730.19]	1647.88*** [1579.33,1716.4]	190.57*** [180.49,200.66]	365.31*** [351.09,379.53]	449.08*** [428.31,469.84]	6.47*** [5.65,7.29]	5.37*** [4.51,6.23]	5.48*** [4.57,6.39]
**Pre-operative benzodiazepine fills**									
Less than 3	Ref	Ref	Ref	Ref	Ref	Ref	Ref	Ref	Ref
3 or more	106.80*** [77.15,136.45]	242.32*** [192.22,292.43]	545.56*** [407.54,683.58]	54.59*** [35.94,73.24]	116.08*** [87.76,144.40]	131.72*** [89.96,173.48]	1.75* [0.24,3.27]	4.42*** [2.70,6.13]	3.90*** [2.07,5.73]
**Comorbidities**									
Mental health conditions	33.50*** [23.88,43.13]	80.39*** [61.36,99.42]	197.89*** [138.35,257.43]	17.02*** [10.96,23.07]	41.38*** [30.62,52.13]	64.35*** [46.33,82.37]	1.20*** [0.70,1.69]	1.73*** [1.08,2.38]	1.33*** [0.54,2.12]

© 2025 Thirukumaran CP et al. *JAMA Network Open*

	Total MME			MME per script			MME per day		
	7 days	*8-to-30 days*	*31-to-90 days*	*7 days*	*8-to-30 days*	*31-to-90 days*	*7 days*	*8-to-30 days*	*31-to-90 days*
	Estimate [95% CI]	*Estimate [95% CI]*	*Estimate [95% CI]*	*Estimate [95% CI]*	*Estimate [95% CI]*	*Estimate [95% CI]*	*Estimate [95% CI]*	*Estimate [95% CI]*	*Estimate [95% CI]*
Alcohol use disorder	-26.54 [-60.58,7.50]	-31.91 [-97.61,33.80]	-198.61 [-402.76,5.54]	-10.72 [-32.13,10.69]	-16.21 [-53.34,20.92]	-63.62* [-125.40,-1.84]	-0.97 [-2.71,0.77]	0.04 [-2.21,2.29]	1.02 [-1.69,3.73]
Tobacco use disorder	34.31*** [14.63,53.99]	83.78*** [46.04,121.53]	248.93*** [135.49,362.38]	31.16*** [18.77,43.54]	46.52*** [25.18,67.86]	84.53*** [50.18,118.87]	1.83*** [0.82,2.83]	1.20 [-0.10,2.49]	1.72* [0.21,3.22]
**Elixhauser's comorbidities**									
Acquired Immunodeficiency Syndrome	-62.42 [-340.44,215.60]	1381.80*** [837.37,1926.24]	2548.49** [1028.91,4068.08]	-106.72 [-281.58,68.13]	1486.66*** [1178.97,1794.36]	1497.67*** [1038.02,1957.32]	-14.20 [-28.41,0.01]	42.60*** [23.95,61.26]	44.93*** [24.76,65.10]
Blood loss anemia	-7.59 [-70.50,55.31]	1.65 [-117.03,120.33]	237.78 [-140.46,616.03]	-15.78 [-55.36,23.81]	25.10 [-42.14,92.33]	59.36 [-55.55,174.27]	-2.58 [-5.79,0.64]	2.69 [-1.39,6.77]	5.56* [0.51,10.62]
Cardiac arrhythmias	-2.17 [-15.06,10.71]	-0.58 [-27.00,25.85]	-32.04 [-115.54,51.46]	-4.86 [-12.97,3.24]	-9.39 [-24.32,5.54]	-16.72 [-41.98,8.54]	-0.20 [-0.86,0.46]	0.05 [-0.86,0.95]	-0.16 [-1.27,0.95]
Chronic pulmonary disease	15.15* [2.62,27.68]	59.73*** [35.27,84.18]	162.72*** [86.69,238.75]	7.46 [-0.42,15.34]	28.44*** [14.62,42.27]	35.85** [12.83,58.86]	0.33 [-0.31,0.97]	1.20** [0.36,2.04]	0.86 [-0.15,1.87]
Coagulopathy	25.08 [-1.56,51.71]	6.44 [-50.03,62.92]	2.98 [-174.34,180.30]	14.06 [-2.69,30.81]	10.45 [-21.47,42.37]	-15.24 [-68.90,38.43]	-0.72 [-2.08,0.64]	1.26 [-0.67,3.20]	-0.26 [-2.62,2.10]
Congestive heart failure	-14.69 [-43.74,14.36]	-7.10 [-66.06,51.86]	62.04 [-115.22,239.29]	-13.15 [-31.42,5.12]	5.03 [-28.29,38.35]	39.60 [-14.02,93.23]	-0.76 [-2.24,0.73]	-0.20 [-2.22,1.82]	0.41 [-1.94,2.77]
Deficiency anemia	-6.11 [-50.60,38.39]	-30.49 [-118.27,57.30]	19.81 [-239.73,279.34]	-0.08 [-28.07,27.92]	-7.39 [-57.02,42.25]	49.40 [-29.20,127.99]	-3.15** [-5.42,-0.87]	-2.32 [-5.33,0.69]	-1.03 [-4.48,2.42]
Diabetes, Complicated	-17.92 [-43.18,7.34]	19.52 [-29.34,68.38]	-16.91 [-170.33,136.51]	-11.89 [-27.78,4.00]	-0.41 [-28.03,27.21]	2.80 [-43.65,49.24]	0.02 [-1.27,1.31]	-0.08 [-1.76,1.59]	0.32 [-1.72,2.35]
Diabetes, uncomplicated	7.52 [-5.59,20.63]	-8.51 [-33.94,16.92]	-34.23 [-113.49,45.02]	1.68 [-6.57,9.92]	-5.14 [-19.51,9.24]	5.50 [-18.50,29.51]	-0.02 [-0.69,0.65]	-0.09 [-0.97,0.78]	-0.03 [-1.09,1.02]
Fluid and electrolyte disorders	12.60 [-4.04,29.24]	-2.59 [-38.21,33.03]	-20.57 [-130.27,89.12]	9.86 [-0.61,20.33]	6.65 [-13.51,26.80]	4.93 [-28.33,38.20]	-0.19 [-1.04,0.66]	0.02 [-1.20,1.24]	-0.49 [-1.95,0.98]
Hypertension, complicated	-10.52 [-42.45,21.41]	-6.72 [-70.23,56.79]	-87.08 [-282.24,108.07]	10.53 [-9.56,30.62]	22.67 [-13.24,58.59]	-35.33 [-94.41,23.75]	0.24 [-1.39,1.88]	1.05 [-1.13,3.23]	-1.61 [-4.20,0.98]
Hypertension, uncomplicated	7.09 [-2.27,16.45]	-7.59 [-26.79,11.61]	-26.14 [-87.83,35.56]	7.28* [1.40,13.17]	0.83 [-10.02,11.69]	-4.97 [-23.65,13.71]	0.09 [-0.39,0.57]	-0.40 [-1.06,0.26]	-1.63*** [-2.45,-0.81]

© 2025 Thirukumaran CP et al. *JAMA Network Open*

	Total MME			MME per script			MME per day		
	7 days	*8-to-30 days*	*31-to-90 days*	*7 days*	*8-to-30 days*	*31-to-90 days*	*7 days*	*8-to-30 days*	*31-to-90 days*
	Estimate [95% CI]	*Estimate [95% CI]*	*Estimate [95% CI]*	*Estimate [95% CI]*	*Estimate [95% CI]*	*Estimate [95% CI]*	*Estimate [95% CI]*	*Estimate [95% CI]*	*Estimate [95% CI]*
Hypothyroidism	13.53* [2.47,24.59]	9.97 [-12.53,32.48]	22.99 [-48.40,94.39]	6.69 [-0.27,13.65]	0.11 [-12.61,12.83]	7.37 [-14.24,28.99]	0.29 [-0.27,0.86]	0.33 [-0.44,1.11]	-0.33 [-1.28,0.62]
Liver disease	13.60 [-26.83,54.04]	56.29 [-23.25,135.82]	212.54 [-27.62,452.69]	-6.97 [-32.40,18.46]	45.80* [0.86,90.75]	72.84* [0.18,145.51]	-1.96 [-4.03,0.10]	0.28 [-2.45,3.00]	0.85 [-2.34,4.04]
Obesity	15.55** [4.75,26.35]	3.98 [-18.06,26.01]	-2.22 [-71.67,67.24]	12.20*** [5.41,19.00]	13.79* [1.31,26.28]	13.81 [-7.35,34.97]	1.45*** [0.90,2.00]	0.70 [-0.06,1.46]	0.82 [-0.11,1.75]
Other neurological disorders	-47.09** [-81.21,-12.97]	-12.93 [-83.60,57.73]	-66.72 [-287.55,154.11]	-16.57 [-38.03,4.89]	-9.77 [-49.70,30.16]	-35.01 [-101.81,31.79]	-2.41** [-4.16,-0.67]	-1.95 [-4.37,0.48]	-3.37* [-6.30,-0.44]
Paralysis	-12.02 [-248.10,224.06]	-21.50 [-386.62,343.62]	423.23 [-934.97,1781.42]	-11.29 [-159.76,137.17]	-116.19 [-322.46,90.08]	127.67 [-283.07,538.42]	-12.75* [-24.82,-0.69]	-10.94 [-23.44,1.57]	0.19 [-17.83,18.21]
Peptic ulcer disease excluding bleeding	-31.77 [-106.24,42.70]	78.85 [-66.44,224.15]	149.18 [-263.39,561.75]	-27.49 [-74.33,19.36]	6.72 [-75.42,88.86]	95.44 [-29.41,220.28]	-2.31 [-6.11,1.50]	-2.50 [-7.48,2.48]	0.98 [-4.50,6.46]
Peripheral vascular disorders	7.27 [-19.44,33.97]	12.24 [-43.53,68.01]	43.99 [-130.65,218.62]	2.28 [-14.51,19.08]	18.61 [-12.90,50.13]	30.13 [-22.70,82.96]	0.13 [-1.23,1.50]	-0.23 [-2.14,1.69]	1.10 [-1.21,3.42]
Pulmonary circulatory disorders	4.27 [-44.73,53.27]	-48.18 [-142.99,46.63]	81.96 [-204.81,368.73]	0.30 [-30.52,31.12]	1.47 [-52.12,55.05]	69.55 [-17.21,156.31]	0.07 [-2.43,2.58]	0.27 [-2.98,3.51]	2.46 [-1.35,6.27]
Renal failure	6.81 [-25.41,39.04]	-19.75 [-83.93,44.42]	-64.86 [-262.26,132.54]	-6.73 [-27.00,13.54]	-21.29 [-57.58,14.99]	1.07 [-58.69,60.84]	-0.19 [-1.84,1.45]	-0.43 [-2.63,1.77]	-1.80 [-4.42,0.82]
Rheumatoid arthritis / collagen vascular diseases	25.91* [4.52,47.31]	86.83*** [45.37,128.29]	254.51*** [132.20,376.81]	21.00** [7.54,34.45]	62.63*** [39.20,86.07]	142.26*** [105.25,179.28]	0.54 [-0.56,1.63]	1.00 [-0.42,2.42]	0.83 [-0.80,2.45]
Valvular disease	-12.69 [-31.28,5.90]	-1.14 [-40.85,38.57]	-27.96 [-155.31,99.40]	-9.81 [-21.50,1.88]	-1.21 [-23.65,21.24]	-3.29 [-41.82,35.24]	-0.35 [-1.30,0.60]	-0.63 [-1.99,0.73]	-0.36 [-2.06,1.33]
Weight loss	63.12 [-25.98,152.23]	37.27 [-133.50,208.05]	126.00 [-340.02,592.01]	35.62 [-20.97,92.21]	2.11 [-97.29,101.51]	29.07 [-116.36,174.50]	1.69 [-2.91,6.28]	-1.60 [-7.66,4.46]	-0.70 [-7.20,5.80]

© 2025 Thirukumaran CP et al. *JAMA Network Open*

	Total MME			MME per script			MME per day		
	7 days	*8-to-30 days*	*31-to-90 days*	*7 days*	*8-to-30 days*	*31-to-90 days*	*7 days*	*8-to-30 days*	*31-to-90 days*
	Estimate [95% CI]	*Estimate [95% CI]*	*Estimate [95% CI]*	*Estimate [95% CI]*	*Estimate [95% CI]*	*Estimate [95% CI]*	*Estimate [95% CI]*	*Estimate [95% CI]*	*Estimate [95% CI]*
**Hospital ownership**									
Private for-profit	Ref	Ref	Ref	Ref	Ref	Ref	Ref	Ref	Ref
Private not-for-profit	89.61** [33.95,145.27]	55.83 [-6.52,118.18]	79.00 [-53.53,211.53]	60.63* [14.20,107.06]	32.05 [-15.45,79.54]	19.72 [-32.85,72.28]	3.09 [-0.47,6.65]	4.06** [0.97,7.14]	2.94* [0.19,5.68]
Government	10.56 [-61.45,82.58]	28.03 [-54.24,110.29]	-59.89 [-235.43,115.65]	-1.70 [-60.88,57.48]	12.89 [-49.12,74.91]	-11.78 [-80.85,57.29]	1.78 [-2.78,6.34]	2.90 [-1.11,6.91]	1.40 [-2.19,4.99]
**Hospital location**									
Rural	Ref	Ref	Ref	Ref	Ref	Ref	Ref	Ref	Ref
Urban	-6.95 [-81.66,67.75]	-63.19 [-152.20,25.83]	-122.27 [-335.34,90.79]	-18.22 [-80.03,43.59]	-36.55 [-102.83,29.73]	-65.51 [-144.43,13.41]	1.48 [-3.27,6.23]	-1.52 [-5.80,2.77]	0.93 [-3.09,4.95]
**Number of beds**									
Small (<200)	Ref	Ref	Ref	Ref	Ref	Ref	Ref	Ref	Ref
Medium (>=200 & <400)	-23.83 [-67.81,20.15]	-12.44 [-59.83,34.94]	13.26 [-83.46,109.97]	-23.83 [-61.05,13.38]	-1.17 [-38.33,35.99]	9.98 [-29.58,49.54]	-2.51 [-5.36,0.34]	-1.45 [-3.87,0.97]	0.46 [-1.65,2.57]
Large (>=400)	-29.43 [-92.34,33.49]	-1.96 [-67.62,63.69]	74.61 [-57.17,206.39]	-32.11 [-86.01,21.79]	-3.43 [-55.97,49.10]	1.99 [-53.06,57.03]	-2.81 [-6.93,1.31]	-2.46 [-5.90,0.98]	-1.53 [-4.49,1.44]
**Medical school affiliation**									
No	Ref	Ref	Ref	Ref	Ref	Ref	Ref	Ref	Ref
Yes	2.52 [-43.80,48.84]	27.79 [-22.58,78.16]	56.36 [-49.77,162.48]	-6.19 [-45.01,32.63]	10.88 [-28.14,49.90]	19.53 [-22.94,61.99]	0.30 [-2.68,3.28]	-0.07 [-2.61,2.47]	0.01 [-2.23,2.25]
**Disproportionate patient mix**	-72.96 [-178.96,33.03]	-146.37* [-276.46,-16.27]	-168.40 [-455.16,118.35]	-21.9 [-106.86,63.07]	-67.83 [-163.14,27.48]	-32.39 [-141.72,76.94]	-12.81*** [-19.38,-6.23]	-14.38*** [-20.51,-8.25]	-12.01*** [-17.59,-6.42]
**Transfer-adjusted casemix index**	203.15*** [113.26,293.05]	115.61* [19.06,212.16]	139.74 [-52.33,331.81]	176.94*** [101.06,252.82]	112.53** [36.44,188.62]	84.61* [4.06,165.16]	8.12** [2.30,13.93]	3.68 [-1.28,8.64]	3.85 [-0.48,8.17]

© 2025 Thirukumaran CP et al. *JAMA Network Open*

	**Total MME**			**MME per script**			**MME per day**		
	7 days	*8-to-30 days*	*31-to-90 days*	*7 days*	*8-to-30 days*	*31-to-90 days*	*7 days*	*8-to-30 days*	*31-to-90 days*
	Estimate [95% CI]	*Estimate [95% CI]*	*Estimate [95% CI]*	*Estimate [95% CI]*	*Estimate [95% CI]*	*Estimate [95% CI]*	*Estimate [95% CI]*	*Estimate [95% CI]*	*Estimate [95% CI]*
**Annual TJR volume**									
Quartile 1	Ref	Ref	Ref	Ref	Ref	Ref	Ref	Ref	Ref
Quartile 2	32.01 [-17.94,81.96]	43.24 [-46.12,132.59]	49.86 [-204.20,303.93]	27.84 [-5.61,61.28]	15.72 [-37.62,69.06]	-15.21 [-94.82,64.40]	3.60** [0.91,6.28]	3.30* [0.02,6.58]	1.45 [-2.16,5.06]
Quartile 3	18.36 [-33.74,70.46]	53.72 [-35.25,142.69]	60.03 [-188.41,308.46]	20.39 [-15.03,55.81]	9.77 [-44.50,64.05]	-18.84 [-98.09,60.41]	3.67* [0.83,6.50]	6.48*** [3.13,9.83]	2.65 [-0.98,6.29]
Quartile 4	-5.68 [-59.91,48.55]	47.37 [-44.27,139.00]	2.85 [-250.72,256.41]	-1.85 [-38.75,35.05]	4.68 [-51.61,60.97]	-25.82 [-107.49,55.86]	1.51 [-1.45,4.46]	6.59*** [3.11,10.07]	3.02 [-0.74,6.79]
**N**	57,098	36,462	24,713	57,098	36,462	24,713	57,098	36,462	24,713

* p<0.05, ** p<0.01, *** p<0.001

Abbreviations: CI: Confidence Interval, Ref: Reference group, N: Number, TJR: Total joint replacement

Notes: Beta estimates from multivariable hierarchical linear regression models with difference-in-differences estimation that control for patient- and hospital-level covariates and hospital-level random effects.

© 2025 Thirukumaran CP et al. *JAMA Network Open*

eTable 3: Estimates from multivariable hierarchical regression models examining the association of Section 3331 with opioid fills in the post-TJR period

	**At least one opioid fill**			**Number of fills**		
	7 days	*8-to-30 days*	*31-to-90 days*	*7 days*	*8-to-30 days*	*31-to-90 days*
	Odds Ratio [95% CI]	*Odds Ratio [95% CI]*	*Odds Ratio [95% CI]*	*Estimate [95% CI]*	*Estimate [95% CI]*	*Estimate [95% CI]*
**Phase**						
Before	Ref	Ref	Ref	Ref	Ref	Ref
After	0.69*** [0.66,0.72]	0.85*** [0.82,0.88]	0.71*** [0.68,0.73]	-0.02*** [-0.03,-0.01]	-0.02* [-0.04,-0.00]	-0.09*** [-0.12,-0.06]
**State**						
CA	Ref	Ref	Ref	Ref	Ref	Ref
NY	1.64*** [1.28,2.12]	0.68*** [0.61,0.76]	0.64*** [0.57,0.71]	-0.05* [-0.09,-0.01]	-0.07** [-0.11,-0.03]	-0.17*** [-0.23,-0.11]
**Phase#State**						
After#NY	1.09* [1.02,1.17]	0.98 [0.92,1.05]	1.04 [0.97,1.12]	0.02** [0.01,0.04]	0.04* [0.01,0.07]	0.15*** [0.09,0.21]
Age	0.98*** [0.98,0.98]	0.96*** [0.96,0.96]	0.97*** [0.97,0.97]	-0.00*** [-0.00,-0.00]	-0.01*** [-0.01,-0.01]	-0.02*** [-0.02,-0.01]
**Gender**						
Male	Ref	Ref	Ref	Ref	Ref	Ref
Female	1.00 [0.97,1.03]	1.13*** [1.09,1.16]	1.18*** [1.14,1.22]	0.01 [-0.00,0.01]	0.01 [-0.00,0.02]	0.01 [-0.02,0.04]
**Race/ethnicity**						
White	Ref	Ref	Ref	Ref	Ref	Ref
Asian	0.96 [0.86,1.06]	0.82*** [0.74,0.91]	0.86* [0.77,0.97]	-0.02 [-0.05,0.01]	-0.07** [-0.12,-0.02]	-0.07 [-0.16,0.02]
Black	0.94 [0.85,1.03]	1.16** [1.06,1.26]	1.48*** [1.34,1.62]	-0.02 [-0.05,0.00]	-0.04* [-0.08,-0.00]	0.02 [-0.05,0.09]
Hispanic	0.97 [0.87,1.08]	0.92 [0.83,1.02]	1.10 [0.99,1.22]	-0.02 [-0.04,0.01]	-0.06* [-0.10,-0.01]	-0.12** [-0.19,-0.04]
North American Native	0.96 [0.68,1.35]	1.01 [0.73,1.41]	1.32 [0.94,1.87]	0.07 [-0.02,0.16]	-0.02 [-0.16,0.12]	0.03 [-0.20,0.25]

© 2025 Thirukumaran CP et al. *JAMA Network Open*

	At least one opioid fill			Number of fills		
	7 days	*8-to-30 days*	*31-to-90 days*	*7 days*	*8-to-30 days*	*31-to-90 days*
	Odds Ratio [95% CI]	*Odds Ratio [95% CI]*	*Odds Ratio [95% CI]*	*Estimate [95% CI]*	*Estimate [95% CI]*	*Estimate [95% CI]*
Unknown/other	0.97 [0.90,1.04]	0.85*** [0.80,0.91]	0.92* [0.85,0.99]	0.00 [-0.02,0.02]	-0.02 [-0.05,0.02]	-0.09** [-0.15,-0.02]
**Dual-eligibility**						
Non-dual-eligible	Ref	Ref	Ref	Ref	Ref	Ref
Dual-eligible	1.22*** [1.15,1.30]	1.18*** [1.11,1.25]	1.35*** [1.27,1.44]	-0.02* [-0.03,-0.00]	-0.03** [-0.06,-0.01]	0.10*** [0.06,0.14]
**Surgery**						
Knee replacement	Ref	Ref	Ref	Ref	Ref	Ref
Hip replacement	0.76*** [0.73,0.78]	0.28*** [0.27,0.29]	0.36*** [0.34,0.37]	-0.09*** [-0.10,-0.09]	-0.20*** [-0.21,-0.18]	-0.26*** [-0.29,-0.24]
**Discharge destination**						
Home	Ref	Ref	Ref	Ref	Ref	Ref
Home with home healthcare	1.19*** [1.15,1.24]	1.11*** [1.07,1.15]	1.08*** [1.04,1.13]	0.01** [0.00,0.02]	0.02* [0.00,0.04]	0.08*** [0.05,0.11]
**Pre-operative opioid fills**						
Less than 3	Ref	Ref	Ref	Ref	Ref	Ref
3 or more	0.96 [0.91,1.02]	4.31*** [4.08,4.55]	9.07*** [8.57,9.59]	0.10*** [0.08,0.11]	0.25*** [0.23,0.27]	0.76*** [0.73,0.79]
**Pre-operative benzodiazepine fills**						
Less than 3	Ref	Ref	Ref	Ref	Ref	Ref
3 or more	1.02 [0.92,1.14]	1.50*** [1.35,1.66]	1.90*** [1.71,2.12]	0.05*** [0.02,0.07]	0.10*** [0.06,0.14]	0.30*** [0.24,0.37]
**Comorbidities**						
Mental health conditions	1.04* [1.01,1.08]	1.26*** [1.22,1.30]	1.40*** [1.35,1.45]	0.03*** [0.02,0.04]	0.05*** [0.03,0.06]	0.09*** [0.07,0.12]
Alcohol use disorder	0.99 [0.88,1.12]	1.08 [0.96,1.21]	1.05 [0.92,1.19]	0.00 [-0.03,0.03]	0.00 [-0.05,0.05]	0.04 [-0.05,0.14]
Tobacco use disorder	1.09* [1.02,1.17]	1.30*** [1.22,1.39]	1.41*** [1.31,1.51]	-0.01 [-0.03,0.01]	0.04** [0.01,0.07]	0.11*** [0.05,0.16]
**Elixhauser's comorbidities**						

© 2025 Thirukumaran CP et al. *JAMA Network Open*

	At least one opioid fill			Number of fills		
	7 days	*8-to-30 days*	*31-to-90 days*	*7 days*	*8-to-30 days*	*31-to-90 days*
	Odds Ratio [95% CI]	*Odds Ratio [95% CI]*	*Odds Ratio [95% CI]*	*Estimate [95% CI]*	*Estimate [95% CI]*	*Estimate [95% CI]*
Acquired Immunodeficiency Syndrome	1.27 [0.46,3.48]	1.23 [0.46,3.30]	1.32 [0.46,3.77]	0.13 [-0.11,0.37]	-0.04 [-0.46,0.38]	-0.41 [-1.09,0.28]
Blood loss anemia	0.79* [0.64,0.98]	1.14 [0.93,1.41]	1.09 [0.87,1.38]	0.01 [-0.05,0.06]	-0.06 [-0.15,0.03]	0.05 [-0.12,0.22]
Cardiac arrhythmias	0.97 [0.93,1.01]	1.03 [0.99,1.08]	1.10*** [1.05,1.16]	0.00 [-0.01,0.01]	0.01 [-0.01,0.03]	-0.01 [-0.04,0.03]
Chronic pulmonary disease	1.02 [0.97,1.06]	1.16*** [1.11,1.21]	1.18*** [1.13,1.24]	0.01 [-0.01,0.02]	0.02* [0.00,0.04]	0.05** [0.02,0.09]
Coagulopathy	0.95 [0.86,1.05]	1.07 [0.97,1.17]	1.11* [1.00,1.23]	0.00 [-0.02,0.03]	-0.01 [-0.06,0.03]	0.01 [-0.07,0.09]
Congestive heart failure	0.96 [0.87,1.06]	0.97 [0.87,1.07]	1.04 [0.93,1.16]	0.00 [-0.03,0.02]	-0.01 [-0.06,0.03]	0.01 [-0.07,0.09]
Deficiency anemia	0.99 [0.84,1.16]	1.04 [0.89,1.21]	1.18 [1.00,1.39]	-0.02 [-0.06,0.02]	-0.02 [-0.09,0.05]	0.00 [-0.12,0.11]
Diabetes, Complicated	1.02 [0.94,1.12]	1.09 [1.00,1.18]	1.08 [0.99,1.19]	0.01 [-0.01,0.03]	0.05* [0.01,0.08]	0.00 [-0.07,0.07]
Diabetes, uncomplicated	0.98 [0.94,1.03]	1.09*** [1.04,1.14]	1.13*** [1.08,1.19]	0.01 [-0.01,0.02]	0.00 [-0.02,0.02]	-0.01 [-0.04,0.03]
Fluid and electrolyte disorders	0.95 [0.90,1.02]	0.99 [0.94,1.05]	1.10** [1.03,1.17]	0.01 [-0.00,0.02]	0.00 [-0.03,0.03]	-0.01 [-0.06,0.04]
Hypertension, complicated	1.07 [0.96,1.20]	1.10 [0.98,1.22]	1.15* [1.02,1.30]	-0.03* [-0.06,-0.00]	-0.01 [-0.06,0.04]	0.02 [-0.06,0.11]
Hypertension, uncomplicated	1.04* [1.00,1.07]	1.02 [0.99,1.05]	1.03 [1.00,1.07]	-0.01* [-0.02,-0.00]	-0.02** [-0.04,-0.01]	-0.03 [-0.05,0.00]
Hypothyroidism	1.03 [0.99,1.07]	1.01 [0.97,1.05]	1.04* [1.00,1.09]	0.01 [-0.00,0.02]	0.01 [-0.01,0.03]	0.02 [-0.02,0.05]
Liver disease	1.02 [0.88,1.18]	1.01 [0.88,1.16]	1.10 [0.95,1.28]	0.01 [-0.02,0.05]	0.02 [-0.04,0.08]	0.10 [-0.01,0.21]
Obesity	1.01 [0.97,1.05]	1.01 [0.97,1.04]	1.03 [0.99,1.07]	0.01* [0.00,0.02]	-0.01 [-0.03,0.01]	-0.03* [-0.06,-0.00]
Other neurological disorders	0.82*** [0.74,0.92]	0.75*** [0.67,0.84]	0.80*** [0.71,0.92]	-0.04** [-0.07,-0.01]	-0.04 [-0.10,0.01]	-0.04 [-0.14,0.06]

© 2025 Thirukumaran CP et al. *JAMA Network Open*

	At least one opioid fill			Number of fills		
	7 days	*8-to-30 days*	*31-to-90 days*	*7 days*	*8-to-30 days*	*31-to-90 days*
	Odds Ratio [95% CI]	*Odds Ratio [95% CI]*	*Odds Ratio [95% CI]*	*Estimate [95% CI]*	*Estimate [95% CI]*	*Estimate [95% CI]*
Paralysis	0.75 [0.36,1.53]	1.93 [0.91,4.13]	0.73 [0.31,1.73]	0.01 [-0.20,0.22]	0.15 [-0.13,0.43]	-0.12 [-0.73,0.49]
Peptic ulcer disease excluding bleeding	1.17 [0.90,1.53]	1.10 [0.85,1.43]	1.41* [1.07,1.86]	0.01 [-0.05,0.08]	0.05 [-0.06,0.16]	0.15 [-0.04,0.33]
Peripheral vascular disorders	1.01 [0.92,1.11]	1.09 [0.99,1.19]	1.12* [1.01,1.24]	-0.01 [-0.03,0.01]	-0.01 [-0.05,0.04]	-0.03 [-0.11,0.05]
Pulmonary circulatory disorders	0.87 [0.73,1.03]	1.10 [0.93,1.30]	1.17 [0.97,1.40]	0.00 [-0.04,0.05]	-0.02 [-0.10,0.05]	-0.01 [-0.14,0.11]
Renal failure	0.97 [0.87,1.09]	0.91 [0.82,1.02]	0.99 [0.87,1.11]	0.02 [-0.01,0.05]	-0.01 [-0.06,0.04]	-0.06 [-0.15,0.03]
Rheumatoid arthritis / collagen vascular diseases	0.89** [0.82,0.95]	1.00 [0.93,1.08]	1.23*** [1.14,1.33]	-0.01 [-0.03,0.01]	0.00 [-0.03,0.03]	-0.04 [-0.09,0.02]
Valvular disease	1.01 [0.95,1.09]	1.07* [1.00,1.14]	1.05 [0.98,1.13]	0.00 [-0.01,0.02]	-0.01 [-0.04,0.03]	0.01 [-0.05,0.07]
Weight loss	0.74* [0.55,0.98]	0.96 [0.72,1.28]	0.95 [0.70,1.29]	0.01 [-0.07,0.09]	0.05 [-0.08,0.18]	-0.10 [-0.31,0.11]
**Hospital ownership**						
Private for-profit	Ref	Ref	Ref	Ref	Ref	Ref
Private not-for-profit	1.14 [0.86,1.51]	0.94 [0.83,1.07]	1.02 [0.91,1.14]	0.06* [0.01,0.10]	0.03 [-0.01,0.07]	0.06 [-0.00,0.12]
Government	0.85 [0.59,1.22]	0.87 [0.74,1.02]	0.94 [0.81,1.09]	0.04 [-0.02,0.10]	0.04 [-0.01,0.10]	-0.03 [-0.10,0.05]
**Hospital location**						
Rural	Ref	Ref	Ref	Ref	Ref	Ref
Urban	1.08 [0.74,1.59]	0.84* [0.70,1.00]	1.16 [0.98,1.37]	0.02 [-0.04,0.08]	-0.01 [-0.07,0.05]	0.04 [-0.05,0.14]
**Number of beds**						
Small (<200)	Ref	Ref	Ref	Ref	Ref	Ref
Medium (>=200 & <400)	0.74** [0.59,0.93]	1.02 [0.92,1.12]	0.97 [0.90,1.06]	0.01 [-0.03,0.04]	-0.02 [-0.05,0.01]	-0.03 [-0.07,0.01]
Large (>=400)	0.90 [0.65,1.26]	1.19* [1.04,1.37]	0.96 [0.85,1.08]	0.02 [-0.03,0.07]	-0.01 [-0.05,0.03]	-0.01 [-0.07,0.05]

© 2025 Thirukumaran CP et al. *JAMA Network Open*

	At least one opioid fill			Number of fills		
	7 days	*8-to-30 days*	*31-to-90 days*	*7 days*	*8-to-30 days*	*31-to-90 days*
	Odds Ratio [95% CI]	*Odds Ratio [95% CI]*	*Odds Ratio [95% CI]*	*Estimate [95% CI]*	*Estimate [95% CI]*	*Estimate [95% CI]*
**Medical school affiliation**						
No	Ref	Ref	Ref	Ref	Ref	Ref
Yes	0.88 [0.70,1.12]	0.97 [0.88,1.07]	0.91* [0.83,1.00]	0.00 [-0.04,0.04]	0.00 [-0.04,0.03]	-0.01 [-0.06,0.03]
**Disproportionate patient mix**	1.28 [0.77,2.13]	0.68** [0.53,0.88]	1.06 [0.84,1.34]	-0.15** [-0.24,-0.06]	-0.16*** [-0.25,-0.08]	-0.12 [-0.24,0.01]
**Transfer-adjusted casemix index**	1.03 [0.65,1.64]	0.68*** [0.56,0.83]	0.82* [0.69,0.97]	-0.01 [-0.08,0.07]	-0.06 [-0.12,0.01]	-0.07 [-0.15,0.01]
**Annual TJR volume**						
Quartile 1	Ref	Ref	Ref	Ref	Ref	Ref
Quartile 2	1.09 [0.90,1.32]	1.08 [0.93,1.27]	1.09 [0.93,1.29]	-0.01 [-0.06,0.03]	-0.01 [-0.07,0.06]	0.08 [-0.04,0.19]
Quartile 3	1.06 [0.86,1.30]	1.19* [1.02,1.40]	1.06 [0.90,1.25]	-0.02 [-0.07,0.02]	0.03 [-0.04,0.10]	0.11* [0.00,0.23]
Quartile 4	1.04 [0.84,1.29]	1.20* [1.02,1.42]	1.00 [0.84,1.18]	-0.01 [-0.05,0.04]	0.03 [-0.04,0.09]	0.07 [-0.04,0.19]
**N**	92,058	92,058	92,058	57,098	36,462	24,713

* p<0.05, ** p<0.01, *** p<0.001

Abbreviations: CI: Confidence Interval, Ref: Reference group, N: Number, TJR: Total joint replacement

Notes: Odds ratios and beta estimates from multivariable hierarchical logistic or linear regression models with difference-in-differences estimation that control for patient- and hospital-level covariates and hospital-level random effects.

© 2025 Thirukumaran CP et al. *JAMA Network Open*

eTable 4: Estimates from multivariable hierarchical regression models examining the association of Section 3331 with opioid days' supply in the post-TJR period

	**Likelihood of opioid fill longer than 7 days**	**Total days supply**			**Days supply per script**		
	7 days	*7 days*	*8-to-30 days*	*31-to-90 days*	*7 days*	*8-to-30 days*	*31-to-90 days*
	Odds Ratio [95% CI]	*Estimate [95% CI]*	*Estimate [95% CI]*	*Estimate [95% CI]*	*Estimate [95% CI]*	*Estimate [95% CI]*	*Estimate [95% CI]*
**Phase**							
Before	Ref	Ref	Ref	Ref	Ref	Ref	Ref
After	0.57*** [0.54,0.60]	-0.03 [-0.21,0.14]	-1.13*** [-1.41,-0.85]	-2.18*** [-2.83,-1.52]	0.36*** [0.25,0.47]	-0.60*** [-0.78,-0.42]	-0.71*** [-1.02,-0.40]
**State**							
CA	Ref	Ref	Ref	Ref	Ref	Ref	Ref
NY	1.64*** [1.26,2.14]	0.01 [-0.70,0.72]	0.72 [-0.15,1.59]	-0.90 [-2.41,0.60]	0.45 [-0.11,1.00]	1.28*** [0.54,2.03]	0.51 [-0.39,1.41]
**Phase#State**							
After#NY	0.29*** [0.26,0.31]	-1.55*** [-1.82,-1.28]	-1.28*** [-1.81,-0.76]	0.26 [-1.00,1.53]	-1.58*** [-1.76,-1.40]	-1.35*** [-1.68,-1.02]	-0.81** [-1.41,-0.20]
Age	0.99** [0.99,1.00]	-0.04*** [-0.05,-0.03]	-0.10*** [-0.12,-0.08]	-0.20*** [-0.25,-0.14]	0.00 [-0.01,0.00]	0.00 [-0.01,0.02]	0.04** [0.01,0.06]
**Gender**							
Male	Ref	Ref	Ref	Ref	Ref	Ref	Ref
Female	1.04 [1.00,1.08]	0.12 [-0.02,0.26]	0.07 [-0.18,0.33]	0.16 [-0.45,0.76]	0.08 [-0.01,0.17]	0.06 [-0.09,0.22]	0.25 [-0.04,0.54]
**Race/ethnicity**							
White	Ref	Ref	Ref	Ref	Ref	Ref	Ref
Asian	0.80*** [0.70,0.91]	-0.69** [-1.16,-0.22]	-0.71 [-1.53,0.11]	-0.77 [-2.70,1.17]	-0.29 [-0.60,0.01]	0.09 [-0.43,0.61]	0.19 [-0.74,1.12]
Black	0.93 [0.82,1.04]	-0.08 [-0.48,0.32]	0.15 [-0.53,0.82]	0.76 [-0.73,2.25]	0.03 [-0.23,0.29]	0.43 [-0.00,0.86]	0.14 [-0.57,0.85]
Hispanic	1.05 [0.92,1.21]	-0.14 [-0.61,0.33]	-1.16** [-1.91,-0.42]	-3.34*** [-4.95,-1.73]	0.12 [-0.19,0.42]	-0.45 [-0.92,0.02]	-0.50 [-1.27,0.27]
North American Native	1.12 [0.71,1.76]	-0.43 [-1.98,1.11]	1.19 [-1.20,3.58]	4.78 [-0.22,9.78]	-0.67 [-1.68,0.34]	1.23 [-0.28,2.73]	1.02 [-1.37,3.41]

© 2025 Thirukumaran CP et al. *JAMA Network Open*

	Likelihood of opioid fill longer than 7 days	Total days supply			Days supply per script		
	7 days	*7 days*	*8-to-30 days*	*31-to-90 days*	*7 days*	*8-to-30 days*	*31-to-90 days*
	Odds Ratio [95% CI]	*Estimate [95% CI]*	*Estimate [95% CI]*	*Estimate [95% CI]*	*Estimate [95% CI]*	*Estimate [95% CI]*	*Estimate [95% CI]*
Unknown/other	0.94 [0.86,1.03]	-0.14 [-0.44,0.17]	-0.27 [-0.83,0.30]	-1.26 [-2.62,0.10]	-0.12 [-0.31,0.08]	-0.11 [-0.47,0.24]	-0.28 [-0.93,0.37]
**Dual-eligibility**							
Non-dual-eligible	Ref	Ref	Ref	Ref	Ref	Ref	Ref
Dual-eligible	1.16*** [1.08,1.25]	0.42** [0.17,0.68]	1.66*** [1.24,2.07]	4.71*** [3.80,5.63]	0.48*** [0.31,0.65]	1.45*** [1.19,1.71]	1.59*** [1.15,2.03]
**Surgery**							
Knee replacement	Ref	Ref	Ref	Ref	Ref	Ref	Ref
Hip replacement	0.85*** [0.82,0.88]	-1.37*** [-1.51,-1.24]	-1.90*** [-2.17,-1.63]	-2.74*** [-3.38,-2.10]	-0.42*** [-0.51,-0.33]	0.14 [-0.03,0.31]	0.75*** [0.44,1.05]
**Discharge destination**							
Home	Ref	Ref	Ref	Ref	Ref	Ref	Ref
Home with home healthcare	1.09** [1.03,1.14]	0.20* [0.02,0.37]	0.32* [0.01,0.62]	0.74* [0.05,1.44]	0.04 [-0.08,0.15]	0.08 [-0.11,0.28]	-0.10 [-0.45,0.24]
**Pre-operative opioid fills**							
Less than 3	Ref	Ref	Ref	Ref	Ref	Ref	Ref
3 or more	1.63*** [1.52,1.76]	3.64*** [3.40,3.88]	10.47*** [10.14,10.80]	26.17*** [25.50,26.85]	1.92*** [1.76,2.08]	5.22*** [5.01,5.43]	6.57*** [6.24,6.89]
**Pre-operative benzodiazepine fills**							
Less than 3	Ref	Ref	Ref	Ref	Ref	Ref	Ref
3 or more	1.14 [0.99,1.30]	1.04*** [0.59,1.49]	1.88*** [1.22,2.55]	5.83*** [4.46,7.19]	0.40** [0.11,0.69]	0.42* [0.00,0.84]	0.36 [-0.29,1.01]
**Comorbidities**							
Mental health conditions	1.02 [0.98,1.06]	0.41*** [0.27,0.56]	0.93*** [0.68,1.18]	2.64*** [2.05,3.23]	0.14** [0.05,0.24]	0.35*** [0.19,0.50]	0.75*** [0.47,1.03]
Alcohol use disorder	1.14 [0.98,1.32]	0.14 [-0.37,0.65]	-0.41 [-1.28,0.46]	-1.99 [-4.02,0.03]	0.13 [-0.20,0.47]	-0.30 [-0.85,0.25]	-1.10* [-2.07,-0.14]

© 2025 Thirukumaran CP et al. *JAMA Network Open*

	Likelihood of opioid fill longer than 7 days	Total days supply			Days supply per script		
	7 days	*7 days*	*8-to-30 days*	*31-to-90 days*	*7 days*	*8-to-30 days*	*31-to-90 days*
	Odds Ratio [95% CI]	*Estimate [95% CI]*	*Estimate [95% CI]*	*Estimate [95% CI]*	*Estimate [95% CI]*	*Estimate [95% CI]*	*Estimate [95% CI]*
Tobacco use disorder	1.00 [0.91,1.09]	0.14 [-0.16,0.44]	1.11*** [0.61,1.61]	3.03*** [1.91,4.16]	0.14 [-0.05,0.34]	0.54*** [0.23,0.86]	1.04*** [0.50,1.58]
**Elixhauser's comorbidities**							
Acquired Immunodeficiency Syndrome	1.65 [0.47,5.81]	1.08 [-3.11,5.27]	6.23 [-0.99,13.44]	-3.92 [-18.97,11.13]	-0.02 [-2.76,2.72]	6.42** [1.87,10.97]	1.89 [-5.29,9.07]
Blood loss anemia	1.16 [0.88,1.52]	0.22 [-0.73,1.17]	-0.48 [-2.06,1.09]	0.95 [-2.80,4.71]	0.28 [-0.34,0.90]	0.07 [-0.93,1.06]	-1.39 [-3.18,0.41]
Cardiac arrhythmias	0.98 [0.92,1.03]	0.03 [-0.16,0.22]	-0.01 [-0.36,0.34]	-0.84* [-1.67,-0.01]	0.02 [-0.11,0.14]	-0.11 [-0.33,0.11]	-0.25 [-0.64,0.15]
Chronic pulmonary disease	1.03 [0.97,1.08]	0.14 [-0.05,0.33]	0.62*** [0.30,0.94]	1.57*** [0.82,2.32]	0.07 [-0.06,0.19]	0.25* [0.05,0.46]	0.26 [-0.10,0.62]
Coagulopathy	1.12 [1.00,1.25]	0.36 [-0.04,0.76]	-0.33 [-1.08,0.41]	0.04 [-1.72,1.79]	0.24 [-0.02,0.50]	-0.14 [-0.61,0.33]	-0.12 [-0.96,0.72]
Congestive heart failure	0.88 [0.78,1.00]	-0.31 [-0.75,0.13]	-0.42 [-1.20,0.36]	0.10 [-1.66,1.85]	-0.25 [-0.53,0.04]	-0.10 [-0.59,0.40]	0.55 [-0.29,1.39]
Deficiency anemia	0.92 [0.76,1.12]	0.43 [-0.24,1.10]	0.22 [-0.94,1.38]	1.45 [-1.12,4.02]	0.54* [0.10,0.97]	0.54 [-0.19,1.28]	1.02 [-0.21,2.25]
Diabetes, Complicated	1.01 [0.91,1.13]	-0.24 [-0.62,0.14]	0.61 [-0.04,1.25]	-0.31 [-1.83,1.21]	-0.18 [-0.43,0.07]	0.11 [-0.30,0.52]	-0.12 [-0.85,0.61]
Diabetes, uncomplicated	0.98 [0.92,1.04]	0.09 [-0.11,0.28]	-0.08 [-0.42,0.25]	-0.13 [-0.92,0.65]	0.04 [-0.09,0.17]	-0.08 [-0.29,0.13]	0.18 [-0.20,0.55]
Fluid and electrolyte disorders	1.09* [1.01,1.17]	0.32* [0.07,0.57]	0.05 [-0.43,0.52]	-0.10 [-1.19,0.98]	0.21* [0.05,0.37]	0.15 [-0.14,0.45]	0.27 [-0.25,0.79]
Hypertension, complicated	1.03 [0.90,1.18]	0.12 [-0.36,0.61]	0.01 [-0.83,0.86]	0.78 [-1.16,2.71]	0.33* [0.02,0.65]	0.19 [-0.34,0.72]	-0.12 [-1.04,0.81]
Hypertension, uncomplicated	1.05* [1.01,1.09]	0.08 [-0.06,0.22]	0.08 [-0.18,0.33]	0.88** [0.27,1.49]	0.12* [0.02,0.21]	0.19* [0.03,0.35]	0.67*** [0.38,0.96]
Hypothyroidism	1.02 [0.97,1.07]	0.13 [-0.03,0.30]	0.09 [-0.21,0.39]	0.82* [0.12,1.53]	0.05 [-0.06,0.15]	-0.12 [-0.31,0.07]	0.32 [-0.01,0.66]

© 2025 Thirukumaran CP et al. *JAMA Network Open*

	Likelihood of opioid fill longer than 7 days	Total days supply			Days supply per script		
	7 days	*7 days*	*8-to-30 days*	*31-to-90 days*	*7 days*	*8-to-30 days*	*31-to-90 days*
	Odds Ratio [95% CI]	*Estimate [95% CI]*	*Estimate [95% CI]*	*Estimate [95% CI]*	*Estimate [95% CI]*	*Estimate [95% CI]*	*Estimate [95% CI]*
Liver disease	0.97 [0.82,1.16]	0.19 [-0.42,0.80]	0.53 [-0.52,1.58]	2.10 [-0.28,4.48]	0.00 [-0.40,0.39]	0.34 [-0.32,1.01]	0.64 [-0.50,1.77]
Obesity	0.97 [0.92,1.01]	0.09 [-0.07,0.25]	0.00 [-0.30,0.29]	-0.38 [-1.08,0.31]	0.01 [-0.09,0.12]	0.16 [-0.02,0.35]	0.14 [-0.19,0.48]
Other neurological disorders	0.88 [0.76,1.02]	-0.43 [-0.95,0.08]	0.39 [-0.55,1.32]	0.81 [-1.37,3.00]	-0.01 [-0.35,0.33]	0.37 [-0.22,0.96]	0.36 [-0.68,1.41]
Paralysis	3.15* [1.05,9.49]	2.4 [-1.16,5.96]	3.52 [-1.32,8.35]	7.52 [-5.93,20.97]	2.1 [-0.22,4.43]	-0.05 [-3.10,3.00]	2.90 [-3.51,9.32]
Peptic ulcer disease excluding bleeding	1.06 [0.76,1.48]	0.44 [-0.68,1.57]	0.84 [-1.09,2.76]	2.08 [-2.01,6.16]	0.03 [-0.70,0.77]	0.36 [-0.86,1.57]	0.24 [-1.71,2.19]
Peripheral vascular disorders	0.95 [0.85,1.07]	0.2 [-0.21,0.60]	0.63 [-0.11,1.37]	0.30 [-1.43,2.03]	0.19 [-0.08,0.45]	0.69** [0.22,1.15]	0.18 [-0.64,1.01]
Pulmonary circulatory disorders	1.11 [0.89,1.37]	-0.18 [-0.92,0.56]	-0.54 [-1.79,0.72]	0.92 [-1.92,3.76]	-0.03 [-0.51,0.45]	0.10 [-0.69,0.89]	1.57* [0.21,2.92]
Renal failure	0.98 [0.86,1.13]	0.04 [-0.44,0.53]	-0.24 [-1.09,0.61]	0.20 [-1.75,2.16]	-0.09 [-0.41,0.23]	-0.12 [-0.66,0.42]	1.23** [0.29,2.16]
Rheumatoid arthritis / collagen vascular diseases	1.04 [0.95,1.14]	0.25 [-0.08,0.57]	1.45*** [0.90,2.00]	4.42*** [3.21,5.63]	0.22* [0.01,0.43]	1.00*** [0.66,1.35]	3.27*** [2.69,3.84]
Valvular disease	1.03 [0.95,1.12]	-0.05 [-0.33,0.23]	0.00 [-0.53,0.52]	-0.17 [-1.43,1.10]	-0.10 [-0.28,0.08]	-0.01 [-0.34,0.32]	-0.09 [-0.69,0.52]
Weight loss	0.88 [0.59,1.31]	0.18 [-1.16,1.51]	3.19** [0.90,5.48]	3.21 [-1.48,7.89]	-0.06 [-0.94,0.82]	1.17 [-0.31,2.66]	1.72 [-0.59,4.02]
**Hospital ownership**							
Private for-profit	Ref	Ref	Ref	Ref	Ref	Ref	Ref
Private not-for-profit	1.23 [0.92,1.65]	1.09** [0.30,1.88]	-0.13 [-1.05,0.79]	0.25 [-1.24,1.74]	0.47 [-0.14,1.09]	-0.32 [-1.12,0.48]	-0.42 [-1.36,0.51]
Government	0.80 [0.55,1.17]	-0.01 [-1.04,1.02]	-0.29 [-1.50,0.92]	-1.37 [-3.34,0.59]	-0.36 [-1.15,0.43]	-0.54 [-1.59,0.50]	-0.65 [-1.88,0.57]
**Hospital location**							
Rural	Ref	Ref	Ref	Ref	Ref	Ref	Ref

© 2025 Thirukumaran CP et al. *JAMA Network Open*

	Likelihood of opioid fill longer than 7 days	Total days supply			Days supply per script		
	7 days	*7 days*	*8-to-30 days*	*31-to-90 days*	*7 days*	*8-to-30 days*	*31-to-90 days*
	Odds Ratio [95% CI]	*Estimate [95% CI]*	*Estimate [95% CI]*	*Estimate [95% CI]*	*Estimate [95% CI]*	*Estimate [95% CI]*	*Estimate [95% CI]*
Urban	0.89 [0.60,1.32]	-0.26 [-1.32,0.80]	0.02 [-1.28,1.32]	-1.19 [-3.49,1.12]	-0.50 [-1.32,0.33]	-0.05 [-1.16,1.07]	-1.22 [-2.60,0.15]
**Number of beds**							
Small (<200)	Ref	Ref	Ref	Ref	Ref	Ref	Ref
Medium (>=200 & <400)	0.98 [0.77,1.24]	0.28 [-0.34,0.90]	0.13 [-0.58,0.84]	-0.53 [-1.63,0.57]	0.10 [-0.38,0.59]	0.25 [-0.38,0.89]	-0.30 [-1.02,0.41]
Large (>=400)	0.97 [0.69,1.35]	0.17 [-0.72,1.05]	0.57 [-0.42,1.55]	0.53 [-0.98,2.05]	-0.13 [-0.83,0.58]	0.47 [-0.43,1.38]	0.21 [-0.79,1.22]
**Medical school affiliation**							
No	Ref	Ref	Ref	Ref	Ref	Ref	Ref
Yes	0.90 [0.70,1.15]	-0.22 [-0.87,0.44]	0.42 [-0.33,1.17]	0.12 [-1.07,1.31]	-0.20 [-0.71,0.32]	0.08 [-0.59,0.74]	0.16 [-0.60,0.92]
**Disproportionate patient mix**	2.21** [1.27,3.84]	1.13 [-0.39,2.65]	2.59** [0.69,4.48]	5.42*** [2.26,8.58]	2.00*** [0.85,3.15]	3.27*** [1.68,4.85]	4.17*** [2.26,6.09]
**Transfer-adjusted casemix index**	2.61*** [1.61,4.21]	2.37*** [1.10,3.64]	1.15 [-0.30,2.59]	0.23 [-1.99,2.45]	1.92*** [0.92,2.92]	1.37* [0.07,2.67]	0.52 [-0.95,1.98]
**Annual TJR volume**							
Quartile 1	Ref	Ref	Ref	Ref	Ref	Ref	Ref
Quartile 2	1.02 [0.81,1.29]	-0.13 [-0.87,0.62]	-0.19 [-1.40,1.01]	0.46 [-2.09,3.01]	-0.04 [-0.55,0.46]	-0.10 [-0.91,0.71]	-0.21 [-1.48,1.06]
Quartile 3	0.94 [0.74,1.20]	-0.34 [-1.11,0.43]	-0.70 [-1.91,0.51]	-0.24 [-2.75,2.28]	-0.13 [-0.66,0.40]	-0.75 [-1.58,0.09]	-0.69 [-1.97,0.59]
Quartile 4	0.95 [0.74,1.23]	-0.28 [-1.08,0.53]	-0.93 [-2.18,0.32]	-0.99 [-3.57,1.59]	-0.10 [-0.66,0.45]	-0.86 [-1.72,0.01]	-0.92 [-2.24,0.41]
**N**	57,098	57,098	36,462	24,713	57,098	36,462	24,713

* p<0.05, ** p<0.01, *** p<0.001

© 2025 Thirukumaran CP et al. *JAMA Network Open*

Abbreviations: CI: Confidence Interval, Ref: Reference group, N: Number, TJR: Total joint replacement

Notes: Odds ratios and beta estimates from multivariable hierarchical logistic or linear regression models with difference-in-differences estimation that control for patient- and hospital-level covariates and hospital-level random effects.

© 2025 Thirukumaran CP et al. *JAMA Network Open*

eTable 5: Sensitivity and secondary analysis – Adjusted estimates from models examining the association of Section 3331 with endpoints in the discharge to 7-day post-TJR period – Pre-Section 3331 phase ending in July 2016 (instead of June 2016 as in the main analysis)

	**California (CA): Change with Section 3331 implementation**		**New York (NY): Change with Section 3331 implementation**		**Change in NY compared to CA**	
	**Main Analysis**	**Sensitivity Analysis**	**Main Analysis**	**Sensitivity Analysis**	**Main Analysis**	**Sensitivity Analysis**
	Column A	*Column B*	*Column C*	*Column D*	*Column E*	*Column F*
	Estimate (95% CI)	*Estimate (95% CI)*	*Estimate (95% CI)*	*Estimate (95% CI)*	*Estimate (95% CI)*	*Estimate (95% CI)*
**Morphine Milligram Equivalents (MME)**						
MME total (N=58,023)	-135.08*** (-146.62, -123.53)	-133.68*** (-145.06, -122.29)	-178.00*** (-191.98, -164.02)	-176.37*** (-190.09, -162.65)	-42.92*** (-61.04, -24.80)	-42.69*** (-60.50, -24.87)
MME per script (N=58,023)	-41.18*** -48.45, -33.91)	-31.82*** (-39.00, -24.65)	-101.51*** -110.31, -92.70)	-90.65*** (-99.30, -82.00)	-60.33*** -71.74, -48.91)	-58.83*** (-70.06, -47.59)
MME per day (N=58,023)	-5.80*** (-6.39, -5.21)	-5.70*** (-6.28, -5.12)	-5.37*** (-6.09, -4.66)	-5.29*** (-5.99, -4.58)	0.42 (-0.50, 1.35)	0.41 (-0.50, 1.33)
**Opioid fills**						
Likelihood of at least one opioid fill (N=93,519)	-7.76*** (-8.57, -6.96)	-7.65*** (-8.45, -6.85)	-5.27*** (-6.40, -4.13)	-4.96*** (-6.07, -3.85)	2.50*** (1.12, 3.88)	2.69*** (1.34, 4.05)
Number of fills (N=58,023)	-0.02*** (-0.03, -0.01)	-0.02*** (-0.03, -0.01)	0.00 (-0.01, 0.01)	0.00 (-0.01, 0.01)	0.02** (0.01, 0.04)	0.02** (0.00, 0.04)
**Days supply**						
Likelihood of opioid fill longer than 7 days (N=58,023)	-11.42*** (-12.51, -10.32)	-3.19*** (-4.19, -2.18)	-36.19*** (-37.99, -34.38)	-26.42*** (-27.63, -25.20)	-24.77*** (-26.74, -22.80)	-23.23*** (-24.81, -21.65)
Days supply total (N=58,023)	-0.03 (-0.21, 0.14)	0.03*** (-0.15, 0.20)	-1.58*** (-1.79, -1.37)	-1.51*** (-1.72, -1.30)	-1.55*** (-1.82, -1.28)	-1.54*** (-1.81, -1.27)
Days supply per script (N=58,023)	0.36*** (0.25, 0.47)	0.52*** (0.41, 0.63)	-1.22*** (-1.36, -1.08)	-1.04*** (-1.17, -0.90)	-1.58*** (-1.76, -1.40)	-1.55*** (-1.73, -1.38)

*p<0.05; **p<0.01; ***p<0.001.

Abbreviations: CI: Confidence Interval, TJR: Total joint replacement

Notes:

© 2025 Thirukumaran CP et al. *JAMA Network Open*

- This analysis re-estimated the discharge to 7-day post-TJR main models by redefining the pre-Section 3331 phase to end in July 2016 (instead of June 2016). The post-Section 3331 phase started in August 2016 in both the main and sensitivity analysis.
- Columns A (main analysis) and B (sensitivity analysis) represent the change in mean values in CA during Section 3331 implementation in NY.
- Columns C (main analysis) and D (sensitivity analysis) represent the change in mean values in NY with Section 3331 implementation in NY.
- Columns E (main analysis) and F (sensitivity analysis) compare the Section 3331-associated change in NY (Columns C and D) with that in CA (Columns A and B): the difference-in-differences (DID) estimate.

© 2025 Thirukumaran CP et al. *JAMA Network Open*

eTable 6: Sensitivity and secondary analysis – Adjusted estimates from models examining the association of Section 3331 with endpoints in the discharge to 7-day post-TJR period – outpatient total knee replacements included in 2018-2019

	**California (CA): Change with Section 3331 implementation**		**New York (NY): Change with Section 3331 implementation**		**Change in NY compared to CA**	
	**Main Analysis**	**Sensitivity Analysis**	**Main Analysis**	**Sensitivity Analysis**	**Main Analysis**	**Sensitivity Analysis**
	Column A	*Column B*	*Column C*	*Column D*	*Column E*	*Column F*
	Estimate (95% CI)	*Estimate (95% CI)*	*Estimate (95% CI)*	*Estimate (95% CI)*	*Estimate (95% CI)*	*Estimate (95% CI)*
**Morphine Milligram Equivalents (MME)**						
MME total (N=60,224)	-135.08*** (-146.62, -123.53)	-135.66*** (-146.95, -124.38)	-178.00*** (-191.98, -164.02)	-178.50** (-192.11, -164.89)	-42.92*** (-61.04, -24.80)	-42.84*** (-60.41, -25.26)
MME per script (N=60,224)	-41.18*** -48.45, -33.91)	-41.82*** (-48.96, -34.67)	-101.51*** -110.31, -92.70)	-101.91*** (-110.53, -93.29)	-60.33*** -71.74, -48.91)	-60.09*** (-71.22, -48.96)
MME per day (N=60,224)	-5.80*** (-6.39, -5.21)	-5.81*** (-6.39, -5.22)	-5.37*** (-6.09, -4.66)	-5.49*** (-6.19, -4.78)	0.42 (-0.50, 1.35)	0.32 (-0.59, 1.23)
**Opioid fills**						
Likelihood of at least one opioid fill (N=97,423)	-7.76*** (-8.57, -6.96)	-8.00*** (-8.80, -7.19)	-5.27*** (-6.40, -4.13)	-4.89*** (-6.01, -3.77)	2.50*** (1.12, 3.88)	3.10*** (1.74, 4.46)
Number of fills (N=60,224)	-0.02*** (-0.03, -0.01)	-0.02*** (-0.03, -0.01)	0.00 (-0.01, 0.01)	0.00 (-0.01, 0.01)	0.02** (0.01, 0.04)	0.02** (0.01, 0.04)
**Days supply**						
Likelihood of opioid fill longer than 7 days (N=60,224)	-11.42*** (-12.51, -10.32)	-11.20*** (-12.28, -10.12)	-36.19*** (-37.99, -34.38)	-36.52*** (-38.27, -34.77)	-24.77*** (-26.74, -22.80)	-25.31*** (-27.23, -23.39)
Days supply total (N=60,224)	-0.03 (-0.21, 0.14)	-0.03 (-0.20, 0.14)	-1.58*** (-1.79, -1.37)	-1.57*** (-1.78, -1.37)	-1.55*** (-1.82, -1.28)	-1.54*** (-1.81, -1.28)
Days supply per script (N=60,224)	0.36*** (0.25, 0.47)	0.36*** (0.24, 0.47)	-1.22*** (-1.36, -1.08)	-1.21*** (-1.35, -1.08)	-1.58*** (-1.76, -1.40)	-1.57*** (-1.74, -1.39)

*p<0.05; **p<0.01; ***p<0.001.

Abbreviations: CI: Confidence Interval, TJR: Total joint replacement

Notes:

- This analysis re-estimated the discharge to 7-day post-TJR main models by including outpatient TJRs in the 2018-2019 period.

© 2025 Thirukumaran CP et al. *JAMA Network Open*

- Columns A (main analysis) and B (sensitivity analysis) represent the change in mean values in CA during Section 3331 implementation in NY.
- Columns C (main analysis) and D (sensitivity analysis) represent the change in mean values in NY with Section 3331 implementation in NY.
- Columns E (main analysis) and F (sensitivity analysis) compare the Section 3331-associated change in NY (Columns C and D) with that in CA (Columns A and B): the difference-in-differences (DID) estimate.

© 2025 Thirukumaran CP et al. *JAMA Network Open*

eTable 7: Sensitivity and secondary analysis – Adjusted estimates from models examining the association of Section 3331 with endpoints in the discharge to 7-day post-TJR period – models estimated separately for total hip replacement patients

	**California (CA): Change with Section 3331 implementation**		**New York (NY): Change with Section 3331 implementation**		**Change in NY compared to CA**	
	**Main Analysis**	**Sensitivity Analysis**	**Main Analysis**	**Sensitivity Analysis**	**Main Analysis**	**Sensitivity Analysis**
	Column A	*Column B*	*Column C*	*Column D*	*Column E*	*Column F*
	Estimate (95% CI)	*Estimate (95% CI)*	*Estimate (95% CI)*	*Estimate (95% CI)*	*Estimate (95% CI)*	*Estimate (95% CI)*
**Morphine Milligram Equivalents (MME)**						
MME total (N=22,534)	-135.08*** (-146.62, -123.53)	-149.99*** (-169.82, -130.16)	-178.00*** (-191.98, -164.02)	-176.19*** (-197.88, -154.49)	-42.92*** (-61.04, -24.80)	-26.20 (-55.61, 3.22)
MME per script (N=22,534)	-41.18*** -48.45, -33.91)	-112.64*** (-124.66, -100.62)	-101.51*** -110.31, -92.70)	-147.83*** (-161.00, -134.67)	-60.33*** -71.74, -48.91)	-35.20*** (-53.05, -17.35)
MME per day (N=22,534)	-5.80*** (-6.39, -5.21)	-6.31*** (-7.24, -5.38)	-5.37*** (-6.09, -4.66)	-4.75*** (-5.77, -3.72)	0.42 (-0.50, 1.35)	1.56* (0.18, 2.95)
**Opioid fills**						
Likelihood of at least one opioid fill (N=37,839)	-7.76*** (-8.57, -6.96)	-8.58*** (-9.89, -7.27)	-5.27*** (-6.40, -4.13)	-5.20*** (-6.89, -3.51)	2.50*** (1.12, 3.88)	3.38** (1.25, 5.51)
Number of fills (N=22,534)	-0.02*** (-0.03, -0.01)	-0.02* (-0.03, 0.00)	0.00 (-0.01, 0.01)	-0.02* (-0.03, 0.00)	0.02** (0.01, 0.04)	0.00 (-0.02, 0.02)
**Days supply**						
Likelihood of opioid fill longer than 7 days (N=22,534)	-11.42*** (-12.51, -10.32)	-14.93*** (-16.72, -13.13)	-36.19*** (-37.99, -34.38)	-38.80*** (-41.22, -36.37)	-24.77*** (-26.74, -22.80)	-23.87*** (-26.75, -20.99)
Days supply total (N=22,534)	-0.03 (-0.21, 0.14)	0.42** (0.16, 0.69)	-1.58*** (-1.79, -1.37)	-1.08*** (-1.38, -0.79)	-1.55*** (-1.82, -1.28)	-1.51*** (-1.90, -1.11)
Days supply per script (N=22,534)	0.36*** (0.25, 0.47)	0.58*** (0.39, 0.77)	-1.22*** (-1.36, -1.08)	-0.77*** (-0.97, -0.56)	-1.58*** (-1.76, -1.40)	-1.35*** (-1.63, -1.07)

*p<0.05; **p<0.01; ***p<0.001.

Abbreviations: CI: Confidence Interval, TJR: Total joint replacement

Notes:

© 2025 Thirukumaran CP et al. *JAMA Network Open*

- This re-estimated the discharge to 7-day post-TJR main models by limiting the cohort to total hip replacement encounters.
- Columns A (main analysis) and B (sensitivity analysis) represent the change in mean values in CA during Section 3331 implementation in NY.
- Columns C (main analysis) and D (sensitivity analysis) represent the change in mean values in NY with Section 3331 implementation in NY.
- Columns E (main analysis) and F (sensitivity analysis) compare the Section 3331-associated change in NY (Columns C and D) with that in CA (Columns A and B): the difference-in-differences (DID) estimate.

© 2025 Thirukumaran CP et al. *JAMA Network Open*

eTable 8: Sensitivity and secondary analysis – Adjusted estimates from models examining the association of Section 3331 with endpoints in the discharge to 7-day post-TJR period – models estimated separately for total knee replacement patients

	**California (CA): Change with Section 3331 implementation**		**New York (NY): Change with Section 3331 implementation**		**Change in NY compared to CA**	
	**Main Analysis**	**Sensitivity Analysis**	**Main Analysis**	**Sensitivity Analysis**	**Main Analysis**	**Sensitivity Analysis**
	Column A	*Column B*	*Column C*	*Column D*	*Column E*	*Column F*
	Estimate (95% CI)	*Estimate (95% CI)*	*Estimate (95% CI)*	*Estimate (95% CI)*	*Estimate (95% CI)*	*Estimate (95% CI)*
**Morphine Milligram Equivalents (MME)**						
MME total (N=34,564)	-135.08*** (-146.62, -123.53)	-125.62*** (-139.78, -111.45)	-178.00*** (-191.98, -164.02)	-182.14*** (-200.46, -163.83)	-42.92*** (-61.04, -24.80)	-56.52*** (-79.63, -33.42)
MME per script (N=34,564)	-41.18*** -48.45, -33.91)	-9.10 (-18.24, 0.04)	-101.51*** -110.31, -92.70)	-86.59*** (-98.42, -74.76)	-60.33*** -71.74, -48.91)	-77.49*** (-92.41, -62.56)
MME per day (N=34,564)	-5.80*** (-6.39, -5.21)	-5.47*** (-6.23, -4.71)	-5.37*** (-6.09, -4.66)	-6.03*** (-7.01, -5.04)	0.42 (-0.50, 1.35)	-0.55 (-1.80, 0.69)
**Opioid fills**						
Likelihood of at least one opioid fill (N=54,219)	-7.76*** (-8.57, -6.96)	-7.36*** (-8.36, -6.35)	-5.27*** (-6.40, -4.13)	-5.32*** (-6.80, -3.84)	2.50*** (1.12, 3.88)	2.04* (0.26, 3.82)
Number of fills (N=34,564)	-0.02*** (-0.03, -0.01)	-0.03*** (-0.04, -0.02)	0.00 (-0.01, 0.01)	0.01 (-0.01, 0.03)	0.02** (0.01, 0.04)	0.04** (0.02, 0.06)
**Days supply**						
Likelihood of opioid fill longer than 7 days (N=34,564)	-11.42*** (-12.51, -10.32)	-9.39*** (-10.71, -8.08)	-36.19*** (-37.99, -34.38)	-33.90*** (-36.03, -31.78)	-24.77*** (-26.74, -22.80)	-24.51*** (-26.91, -22.11)
Days supply total (N=34,564)	-0.03 (-0.21, 0.14)	-0.04 (-0.27, 0.19)	-1.58*** (-1.79, -1.37)	-1.59*** (-1.88, -1.29)	-1.55*** (-1.82, -1.28)	-1.55*** (-1.92, -1.18)
Days supply per script (N=34,564)	0.36*** (0.25, 0.47)	0.37*** (0.23, 0.52)	-1.22*** (-1.36, -1.08)	-1.30*** (-1.49, -1.12)	-1.58*** (-1.76, -1.40)	-1.68*** (-1.91, -1.44)

*p<0.05; **p<0.01; ***p<0.001.

Abbreviations: CI: Confidence Interval, TJR: Total joint replacement

Notes:

© 2025 Thirukumaran CP et al. *JAMA Network Open*

- This analysis re-estimated the discharge to 7-day post-TJR main models by limiting the cohort to total knee replacement encounters.
- Columns A (main analysis) and B (sensitivity analysis) represent the change in mean values in CA during Section 3331 implementation in NY.
- Columns C (main analysis) and D (sensitivity analysis) represent the change in mean values in NY with Section 3331 implementation in NY.
- Columns E (main analysis) and F (sensitivity analysis) compare the Section 3331-associated change in NY (Columns C and D) with that in CA (Columns A and B): the difference-in-differences (DID) estimate.

© 2025 Thirukumaran CP et al. *JAMA Network Open*

eTable 9: Sensitivity and secondary analysis – Adjusted estimates from models examining the association of Section 3331 with endpoints in the discharge to 7-day post-TJR period – models estimated among opioid naïve patients only.

	**California (CA): Change with Section 3331 implementation**		**New York (NY): Change with Section 3331 implementation**		**Change in NY compared to CA**	
	**Main Analysis**	**Sensitivity Analysis**	**Main Analysis**	**Sensitivity Analysis**	**Main Analysis**	**Sensitivity Analysis**
	Column A	*Column B*	*Column C*	*Column D*	*Column E*	*Column F*
	Estimate (95% CI)	*Estimate (95% CI)*	*Estimate (95% CI)*	*Estimate (95% CI)*	*Estimate (95% CI)*	*Estimate (95% CI)*
**Morphine Milligram Equivalents (MME)**						
MME total (N=39,546)	-135.08*** (-146.62, -123.53)	-51.64*** (-61.05, -42.23)	-178.00*** (-191.98, -164.02)	-109.10*** (-119.09, -99.12)	-42.92*** (-61.04, -24.80)	-57.46*** (-71.17, -43.75)
MME per script (N=39,546)	-41.18*** -48.45, -33.91)	-25.64*** (-32.88, -18.39)	-101.51*** -110.31, -92.70)	-87.04*** (-94.73, -79.34)	-60.33*** -71.74, -48.91)	-61.40*** (-71.97, -50.84)
MME per day (N=39,546)	-5.80*** (-6.39, -5.21)	-5.53*** (-6.24, -4.82)	-5.37*** (-6.09, -4.66)	-5.33*** (-6.08, -4.57)	0.42 (-0.50, 1.35)	0.20 (-0.84, 1.24)
**Opioid fills**						
Likelihood of at least one opioid fill (N=51,970)	-7.76*** (-8.57, -6.96)	-6.75*** (-7.89, -5.60)	-5.27*** (-6.40, -4.13)	-4.61*** (-5.88, -3.33)	2.50*** (1.12, 3.88)	2.14* (0.43, 3.85)
Number of fills (N=39,546)	-0.02*** (-0.03, -0.01)	-0.01 (-0.02, 0.01)	0.00 (-0.01, 0.01)	0.00 (-0.02, 0.01)	0.02** (0.01, 0.04)	0.00 (-0.01, 0.02)
**Days supply**						
Likelihood of opioid fill longer than 7 days (N=39,546)	-11.42*** (-12.51, -10.32)	-13.50*** (-14.90, -12.10)	-36.19*** (-37.99, -34.38)	-37.09*** (-39.06, -35.12)	-24.77*** (-26.74, -22.80)	-23.59*** (-25.83, -21.34)
Days supply total (N=39,546)	-0.03 (-0.21, 0.14)	-0.15 (-0.35, 0.05)	-1.58*** (-1.79, -1.37)	-1.79*** (-2.00, -1.58)	-1.55*** (-1.82, -1.28)	-1.64*** (-1.93, -1.35)
Days supply per script (N=39,546)	0.36*** (0.25, 0.47)	0.13 (0.00, 0.27)	-1.22*** (-1.36, -1.08)	-1.39*** (-1.53, -1.24)	-1.58*** (-1.76, -1.40)	-1.52*** (-1.72, -1.32)

*p<0.05; **p<0.01; ***p<0.001.

Abbreviations: CI: Confidence Interval, TJR: Total joint replacement

© 2025 Thirukumaran CP et al. *JAMA Network Open*

Notes:

- This analysis re-estimated the discharge to 7-day post-TJR main models by limiting the cohort to opioid naïve (patients without an opioid fill in the 90-day period before admission) patients only.
- Columns A (main analysis) and B (sensitivity analysis) represent the change in mean values in CA during Section 3331 implementation in NY.
- Columns C (main analysis) and D (sensitivity analysis) represent the change in mean values in NY with Section 3331 implementation in NY.
- Columns E (main analysis) and F (sensitivity analysis) compare the Section 3331-associated change in NY (Columns C and D) with that in CA (Columns A and B): the difference-in-differences (DID) estimate.

© 2025 Thirukumaran CP et al. *JAMA Network Open*

eTable 10: Sensitivity and secondary analysis – Adjusted estimates from models examining the association of Section 3331 with endpoints in the discharge to 7-day post-TJR period for patients belonging to various race/ethnicity groups

	CA		NY		Change in NY compared to CA	Change with respect to Non-Hispanic White patients
	**Before**	**After**	**Before**	**After**		
	Column A	*Column B*	*Column C*	*Column D*	*Column E=<br>(D-C)-(B-A)*	*Column F*
	Estimate<br>(95% CI)	*Estimate<br>(95% CI)*	*Estimate<br>(95% CI)*	*Estimate<br>(95% CI)*	*Estimate<br>(95% CI)*	*Estimate<br>(95% CI)*
**Morphine Milligram Equivalents (MME)**						
**MME per day (N=53,133)**						
Black	65.12<br>(61.38, 68.87)	61.52<br>(58.26, 64.78)	52.71<br>(48.54, 56.87)	49.50<br>(46.13, 52.87)	0.39<br>(-5.19, 5.98)	-0.18<br>(-5.84, 5.49)
Hispanic	62.85<br>(59.65, 66.05)	55.98<br>(53.17, 58.80)	50.52<br>(43.28, 57.76)	44.72<br>(39.21, 50.23)	1.06<br>(-7.70, 9.83)	0.49<br>(-8.32, 9.31)
White	64.33<br>(62.35, 66.30)	58.39<br>(56.43, 60.35)	54.10<br>(51.31, 56.88)	48.73<br>(45.98, 51.48)	0.57<br>(-0.41, 1.55)	Ref
**MME per script (N=53,133)**						
Black	631.79<br>(584.62, 678.96)	558.38<br>(517.15, 599.62)	549.37<br>(496.51, 602.24)	420.86<br>(377.57, 464.15)	-55.11<br>(-124.45, 14.24)	3.18<br>(-67.11, 73.47)
Hispanic	564.47<br>(523.97, 604.97)	495.09<br>(459.22, 530.97)	528.87<br>(438.37, 619.37)	381.87<br>(312.57, 451.16)	-77.62<br>(-186.45, 31.20)	-19.34<br>(-128.77, 90.10)
White	602.23<br>(576.48, 627.98)	509.61<br>(484.07, 535.15)	553.52<br>(517.17, 589.87)	402.61<br>(366.72, 438.50)	-58.29***<br>(-70.50, -46.08)	Ref
**MME total (N=53,133)**						
Black	773.24<br>(702.72, 843.76)	697.32<br>(637.24, 757.39)	641.45<br>(566.26, 716.63)	491.75<br>(434.31, 549.18)	-73.78<br>(-184.85, 37.30)	-36.91<br>(-149.52, 75.69)
Hispanic	664.99<br>(606.22, 723.76)	590.59<br>(540.14, 641.04)	567.86<br>(428.12, 707.60)	434.80<br>(330.79, 538.81)	-58.66<br>(-233.15, 115.83)	-21.80<br>(-197.28, 153.69)
White	752.37<br>(721.65, 783.10)	610.94<br>(580.67, 641.21)	657.93<br>(614.75, 701.11)	479.63<br>(437.44, 521.82)	-36.86***<br>(-56.42, -17.31)	Ref
**Opioid fills**						
**Likelihood of at least one opioid fill (N=85,689)**						
Black	63.88<br>(58.60, 69.15)	56.91<br>(52.33, 61.49)	67.63<br>(61.38, 73.88)	64.79<br>(59.49, 70.09)	4.12<br>(-3.60, 11.84)	1.63<br>(-6.20, 9.46)

© 2025 Thirukumaran CP et al. *JAMA Network Open*

	CA		NY		Change in NY compared to CA	Change with respect to Non-Hispanic White patients
	**Before**	**After**	**Before**	**After**		
	Column A	*Column B*	*Column C*	*Column D*	*Column E= (D-C)-(B-A)*	*Column F*
	Estimate (95% CI)	*Estimate (95% CI)*	*Estimate (95% CI)*	*Estimate (95% CI)*	*Estimate (95% CI)*	*Estimate (95% CI)*
Hispanic	65.21 (60.53, 69.89)	55.32 (51.08, 59.56)	67.80 (57.56, 78.04)	65.45 (57.40, 73.49)	7.54 (-4.73, 19.81)	5.05 (-7.30, 17.39)
White	62.86 (59.84, 65.89)	55.27 (52.11, 58.43)	72.83 (68.82, 76.84)	67.73 (63.43, 72.03)	2.49** (1.05, 3.94)	Ref
**Number of fills (N=53,133)**						
Black	1.27 (1.21, 1.33)	1.23 (1.18, 1.28)	1.19 (1.12, 1.25)	1.15 (1.10, 1.20)	0.00 (-0.10, 0.09)	-0.03 (-0.12, 0.07)
Hispanic	1.21 (1.16, 1.26)	1.23 (1.19, 1.27)	1.13 (1.01, 1.25)	1.20 (1.11, 1.29)	0.05 (-0.10, 0.20)	0.03 (-0.12, 0.18)
White	1.25 (1.23, 1.28)	1.23 (1.20, 1.25)	1.20 (1.17, 1.24)	1.20 (1.17, 1.24)	0.03** (0.01, 0.04)	Ref
**Days supply**						
**Likelihood of opioid fill longer than 7 days (N=53,133)**						
Black	68.29 (61.91, 74.67)	51.36 (45.63, 57.08)	78.95 (73.02, 84.87)	37.48 (31.82, 43.13)	-24.53*** (-34.07, -14.99)	-0.05 (-9.61, 9.52)
Hispanic	61.79 (56.26, 67.32)	59.60 (54.72, 64.49)	83.42 (72.93, 93.91)	40.53 (31.25, 49.81)	-40.70*** (-54.84, -26.57)	-16.21* (-30.32, -2.11)
White	66.97 (63.88, 70.06)	55.58 (52.19, 58.97)	76.06 (72.40, 79.71)	40.18 (35.44, 44.92)	-24.49*** (-26.56, -22.42)	Ref
**Total days supply (N=53,133)**						
Black	13.47 (12.43, 14.51)	11.93 (11.05, 12.81)	13.18 (12.08, 14.28)	9.57 (8.74, 10.40)	-2.07* (-3.72, -0.43)	-0.61 (-2.28, 1.06)
Hispanic	11.88 (11.02, 12.74)	11.77 (11.04, 12.51)	14.13 (12.07, 16.19)	10.80 (9.27, 12.33)	-3.22* (-5.81, -0.63)	-1.76 (-4.36, 0.84)
White	13.11 (12.67, 13.54)	11.61 (11.18, 12.04)	13.13 (12.52, 13.74)	10.17 (9.57, 10.76)	-1.46*** (-1.75, -1.17)	Ref
**Days supply per script (N=53,133)**						
Black	10.65 (9.95, 11.35)	9.65 (9.04, 10.25)	10.95 (10.19, 11.71)	8.32 (7.72, 8.92)	-1.63** (-2.70, -0.55)	-0.07 (-1.16, 1.02)
Hispanic	9.89 (9.30, 10.48)	9.66 (9.15, 10.18)	12.78 (11.41, 14.15)	9.19 (8.16, 10.22)	-3.37*** (-5.05, -1.68)	-1.81* (-3.50, -0.12)

© 2025 Thirukumaran CP et al. *JAMA Network Open*

	CA		NY		Change in NY compared to CA	Change with respect to Non-Hispanic White patients
	**Before**	**After**	**Before**	**After**		
	Column A	*Column B*	*Column C*	*Column D*	*Column E= (D-C)-(B-A)*	*Column F*
	Estimate (95% CI)	*Estimate (95% CI)*	*Estimate (95% CI)*	*Estimate (95% CI)*	*Estimate (95% CI)*	*Estimate (95% CI)*
White	10.40 (10.06, 10.74)	9.53 (9.19, 9.87)	10.90 (10.42, 11.38)	8.47 (8.00, 8.94)	-1.55*** (-1.74, -1.37)	Ref

*p<0.05; **p<0.01; ***p<0.001.

Abbreviations: CI: Confidence Interval; TJR: Total joint replacement, Ref: Reference group

Notes:

- Columns A and B represent the mean values in CA in the pre- and post-Section 3331 implementation periods, respectively.
- Columns C and D represent the mean values in NY in the pre- and post-Section 3331 implementation periods, respectively.
- Column E represents the change in mean values in NY with Section 3331 implementation in NY compared to the change in CA: the difference-in-differences (DID) estimate.
- Column F represents the Section 3331-associated change for Black and Hispanic patients compared to White patients in NY compared to CA: the triple differences (DDD) estimate.

© 2025 Thirukumaran CP et al. *JAMA Network Open*

eTable 11: Sensitivity and secondary analysis – Adjusted estimates from models examining the association of Section 3331 with endpoints in the discharge to 7-day post-TJR period for dual- and non-dual-eligible patients

	CA		NY		Change in NY compared to CA	Change with respect to White patients
	**Before**	**After**	**Before**	**After**		
	Column A	*Column B*	*Column C*	*Column D*	*Column E= (D-C)-(B-A)*	*Column F*
	Estimate (95% CI)	*Estimate (95% CI)*	*Estimate (95% CI)*	*Estimate (95% CI)*	*Estimate (95% CI)*	*Estimate (95% CI)*
**Morphine Milligram Equivalents (MME)**						
**MME per day (N=57,098)**						
Non-dual-eligible	64.75 (62.79, 66.70)	58.54 (56.60, 60.47)	54.42 (51.64, 57.19)	49.02 (46.29, 51.76)	0.82 (-0.15, 1.79)	Ref
Dual-eligible	58.83 (56.50, 61.16)	56.02 (53.80, 58.23)	51.53 (47.99, 55.07)	46.48 (43.32, 49.64)	-2.23 (-5.50, 1.03)	-3.05 (-6.45, 0.34)
**MME per script (N=57,098)**						
Non-dual-eligible	603.18 (577.70, 628.66)	507.22 (481.93, 532.51)	553.05 (516.80, 589.29)	400.42 (364.63, 436.21)	-56.67*** (-68.58, -44.75)	Ref
Dual-eligible	569.02 (539.09, 598.94)	516.76 (488.27, 545.25)	528.90 (483.65, 574.15)	403.37 (362.64, 444.09)	-73.27*** (-113.48, -33.07)	-16.61 (-58.39, 25.18)
**MME total (N=57,098)**						
Non-dual-eligible	749.34 (718.96, 779.72)	605.75 (575.80, 635.70)	655.78 (612.80, 698.76)	474.79 (432.76, 516.81)	-37.40*** (-56.32, -18.48)	Ref
Dual-eligible	696.77 (657.34, 736.20)	622.69 (586.10, 659.28)	610.21 (549.33, 671.08)	477.46 (425.22, 529.69)	-58.67 (-122.52, 5.18)	-21.27 (-87.63, 45.10)
**Opioid fills**						
**Likelihood of at least one opioid fill (N=92,058)**						
Non-dual-eligible	62.37 (59.36, 65.38)	54.55 (51.42, 57.68)	72.66 (68.65, 76.66)	67.30 (62.99, 71.61)	2.46** (1.02, 3.90)	Ref
Dual-eligible	67.76 (64.43, 71.09)	60.34 (56.97, 63.72)	72.15 (67.08, 77.22)	67.88 (63.09, 72.68)	3.15 (-1.40, 7.71)	0.69 (-4.04, 5.42)
**Number of fills (N=57,098)**						
Non-dual-eligible	1.25 (1.23, 1.28)	1.23 (1.20, 1.25)	1.20 (1.17, 1.24)	1.20 (1.17, 1.24)	0.02* (0.00, 0.04)	Ref
Dual-eligible	1.24 (1.21, 1.28)	1.22 (1.19, 1.25)	1.15 (1.10, 1.20)	1.17 (1.13, 1.22)	0.05 (-0.01, 0.10)	0.03 (-0.03, 0.08)

© 2025 Thirukumaran CP et al. *JAMA Network Open*

	CA		NY		Change in NY compared to CA	Change with respect to White patients
	**Before**	**After**	**Before**	**After**		
	Column A	*Column B*	*Column C*	*Column D*	*Column E= (D-C)-(B-A)*	*Column F*
	Estimate (95% CI)	*Estimate (95% CI)*	*Estimate (95% CI)*	*Estimate (95% CI)*	*Estimate (95% CI)*	*Estimate (95% CI)*
**Days supply**						
**Likelihood of opioid fill longer than 7 days (N=57,098)**						
Non-dual-eligible	66.78 (63.72, 69.83)	55.12 (51.77, 58.48)	75.82 (72.15, 79.48)	39.45 (34.75, 44.14)	-24.72*** (-26.75, -22.70)	Ref
Dual-eligible	69.20 (65.56, 72.85)	59.50 (55.73, 63.27)	75.96 (70.94, 80.99)	42.00 (36.52, 47.49)	-24.25*** (-29.78, -18.71)	0.47 (-5.14, 6.08)
**Total days supply (N=57,098)**						
Non-dual-eligible	13.00 (12.57, 13.43)	11.51 (11.09, 11.93)	13.08 (12.47, 13.69)	10.04 (9.45, 10.64)	-1.55*** (-1.84, -1.27)	Ref
Dual-eligible	13.48 (12.91, 14.05)	12.18 (11.65, 12.71)	12.90 (12.01, 13.79)	10.24 (9.48, 11.00)	-1.37** (-2.33, -0.40)	0.18 (-0.82, 1.19)
**Days supply per script (N=57,098)**						
Non-dual-eligible	10.36 (10.02, 10.70)	9.48 (9.14, 9.81)	10.84 (10.36, 11.31)	8.37 (7.90, 8.84)	-1.58*** (-1.77, -1.40)	Ref
Dual-eligible	10.89 (10.47, 11.30)	10.03 (9.64, 10.43)	11.14 (10.50, 11.78)	8.74 (8.19, 9.30)	-1.54*** (-2.17, -0.91)	0.04 (-0.61, 0.69)

*p<0.05; **p<0.01; ***p<0.001.

Abbreviations: CI: Confidence Interval; TJR: Total joint replacement

Notes:

- Columns A and B represent the mean values in CA in the pre- and post-Section 3331 implementation periods, respectively.
- Columns C and D represent the mean values in NY in the pre- and post-Section 3331 implementation periods, respectively.
- Columns E represents the change in mean values in NY with Section 3331 implementation in NY compared to the change in CA: the difference-in-differences (DID) estimate.
- Columns F represents the Section 3331-associated change for dual-eligible patients compared to non-dual-eligible patients in NY compared to CA: the triple differences (DDD) estimate.

© 2025 Thirukumaran CP et al. *JAMA Network Open*

eTable 12: Sensitivity and secondary analysis – Adjusted estimates from models examining the association of Section 3331 with endpoints in the cumulative 90-day post-TJR period (date of discharge to 90 days following discharge)

	**California (CA)**			**New York (NY)**			**Change in NY compared to CA**
	**Before**	**After**	**Change**	**Before**	**After**	**Change**	
	Column A	*Column B*	*Column C=A-B*	*Column D*	*Column E*	*Column F=E-D*	*Column G=F-C*
	Estimate (95% CI)	*Estimate (95% CI)*	*Estimate (95% CI)*	*Estimate (95% CI)*	*Estimate (95% CI)*	*Estimate (95% CI)*	*Estimate (95% CI)*
**Morphine Milligram Equivalents (MME)**							
MME total (N=72,137)	1586.75 (1526.95, 1646.55)	1227.43 (1170.37, 1284.49)	-359.32*** (-398.33, -320.30)	1295.44 (1205.27, 1385.62)	954.19 (870.05, 1038.33)	-341.25*** (-393.34, -289.16)	18.07 (-46.94, 83.08)
MME per script (N=72,137)	601.71 (579.63, 623.80)	506.90 (485.06, 528.75)	-94.81*** (-102.38, -87.24)	563.63 (530.59, 596.67)	418.48 (386.08, 450.88)	-145.15*** (-155.28, -135.02)	-50.34*** (-62.99, -37.70)
MME per day (N=72,137)	57.89 (56.35, 59.42)	52.02 (50.50, 53.55)	-5.86*** (-6.34, -5.39)	50.31 (48.01, 52.61)	45.08 (42.81, 47.34)	-5.23*** (-5.87, -4.60)	0.63 (-0.16, 1.42)
**Opioid fills**							
Likelihood of at least one opioid fill (N=92,058)	81.44 (79.74, 83.14)	75.11 (73.10, 77.12)	-6.33*** (-7.06, -5.61)	83.67 (81.27, 86.07)	78.70 (75.92, 81.48)	-4.97*** (-5.99, -3.94)	1.36* (0.12, 2.61)
Number of fills (N=72,137)	2.45 (2.40, 2.50)	2.25 (2.20, 2.30)	-0.19*** (-0.22, -0.17)	2.12 (2.05, 2.20)	2.04 (1.97, 2.12)	-0.08*** (-0.12, -0.05)	0.11*** (0.07, 0.16)
**Days supply**							
Days supply total (N=72,137)	30.30 (29.51, 31.10)	26.02 (25.26, 26.79)	-4.28*** (-4.74, -3.83)	27.47 (26.28, 28.67)	21.99 (20.86, 23.12)	-5.48*** (-6.09, -4.87)	-1.20** (-1.96, -0.44)
Days supply per script (N=72,137)	12.03 (11.68, 12.38)	12.05 (11.70, 12.39)	0.02 (-0.10, 0.14)	12.29 (11.77, 12.80)	10.86 (10.35, 11.37)	-1.43*** (-1.59, -1.27)	-1.44*** (-1.64, -1.24)

*p<0.05; **p<0.01; ***p<0.001.

Abbreviations: CI: Confidence Interval, TJR: Total joint replacement

Notes:

- This analysis examined the association of Section 3331 implementation with opioid endpoints in the cumulative 90-day post-TJR period.

© 2025 Thirukumaran CP et al. *JAMA Network Open*

- Columns A and B represent the mean values in CA in the pre- and post-Section 3331 implementation periods, respectively.
- Column C represents the difference in the pre- and post-Section 3331 periods in CA.
- Columns D and E represent the mean values in NY in the pre- and post-Section 3331 implementation periods, respectively.
- Column F represents the difference in the pre- and post-Section 3331 periods in NY
- Column G compares the Section 3331-associated change in NY (Column F) with that in CA (Column C): the difference-in-differences (DID) estimate.

© 2025 Thirukumaran CP et al. *JAMA Network Open*

eTable 13: Sensitivity and secondary analysis – Adjusted estimates from models examining the association of Section 3331 with endpoints in the cumulative 30-day post-TJR period (date of discharge to 30 days following discharge)

	California (CA)			New York (NY)			Change in NY compared to CA
	**Before**	**After**	**Change**	**Before**	**After**	**Change**	
	Column A	*Column B*	*Column C=A-B*	*Column D*	*Column E*	*Column F=E-D*	*Column G=F-C*
	Estimate (95% CI)	*Estimate (95% CI)*	*Estimate (95% CI)*	*Estimate (95% CI)*	*Estimate (95% CI)*	*Estimate (95% CI)*	*Estimate (95% CI)*
**Morphine Milligram Equivalents (MME)**							
MME total (N=69,480)	1101.38 (1062.38, 1140.38)	876.24 (838.06, 914.42)	-225.14*** (-242.98, -207.29)	950.00 (892.08, 1007.91)	685.06 (629.08, 741.03)	-264.94*** (-288.45, -241.43)	-39.80** (-69.30, -10.31)
MME per script (N=69,480)	607.48 (584.14, 630.83)	558.62 (535.48, 581.76)	-48.86*** (-56.08, -41.64)	564.85 (530.24, 599.46)	459.33 (425.26, 493.41)	-105.52*** (-115.03, -96.00)	-56.65*** (-68.60, -44.71)
MME per day (N=69,480)	61.09 (59.41, 62.77)	54.80 (53.13, 56.47)	-6.29*** (-6.79, -5.79)	52.26 (49.77, 54.75)	46.70 (44.24, 49.15)	-5.56*** (-6.22, -4.90)	0.73 (-0.10, 1.56)
**Opioid fills**							
Likelihood of at least one opioid fill (N=92,058)	77.87 (75.92, 79.82)	71.79 (69.58, 74.00)	-6.08*** (-6.82, -5.34)	81.58 (78.91, 84.25)	76.80 (73.78, 79.82)	-4.78*** (-5.83, -3.74)	1.30* (0.02, 2.57)
Number of fills (N=69,480)	1.81 (1.77, 1.85)	1.73 (1.69, 1.77)	-0.08*** (-0.10, -0.06)	1.68 (1.62, 1.74)	1.64 (1.58, 1.70)	-0.04** (-0.06, -0.02)	0.04** (0.01, 0.07)
**Days supply**							
Days supply total (N=69,480)	19.94 (19.41, 20.48)	17.60 (17.08, 18.12)	-2.34*** (-2.59, -2.09)	19.57 (18.77, 20.36)	15.32 (14.56, 16.09)	-4.24*** (-4.57, -3.92)	-1.90*** (-2.32, -1.49)
Days supply per script (N=69,480)	11.19 (10.86, 11.52)	11.37 (11.04, 11.69)	0.17** (0.06, 0.28)	11.67 (11.18, 12.17)	10.26 (9.77, 10.74)	-1.41*** (-1.56, -1.27)	-1.59*** (-1.77, -1.41)

*p<0.05; **p<0.01; ***p<0.001.

Abbreviations: CI: Confidence Interval, TJR: Total joint replacement

Notes:

- This analysis examined the association of Section 3331 implementation with opioid endpoints in the cumulative 30-day post-TJR period.

© 2025 Thirukumaran CP et al. *JAMA Network Open*

- Columns A and B represent the mean values in CA in the pre- and post-Section 3331 implementation periods, respectively.
- Column C represents the difference in the pre- and post-Section 3331 periods in CA.
- Columns D and E represent the mean values in NY in the pre- and post-Section 3331 implementation periods, respectively.
- Column F represents the difference in the pre- and post-Section 3331 periods in NY
- Column G compares the Section 3331-associated change in NY (Column F) with that in CA (Column C): the difference-in-differences (DID) estimate.

© 2025 Thirukumaran CP et al. *JAMA Network Open*

eReferences

1. Grafova IB, Jarrin OF. Beyond Black and White: Mapping Misclassification of Medicare Beneficiaries Race and Ethnicity. *Med Care Res Rev*. Oct 2021;78(5):616-626. doi:10.1177/1077558720935733

2. Bilinski A, Hatfield LA. Nothing to see here? Non-inferiority approaches to parallel trends and other model assumptions. *arXiv preprint arXiv:180503273*. 2018;

3. Nakamoto CH, Cutler DM, Beaulieu ND, Uscher-Pines L, Mehrotra A. The Impact Of Telemedicine On Medicare Utilization, Spending, And Quality, 2019-22. *Health Aff (Millwood)*. May 2024;43(5):691-700. doi:10.1377/hlthaff.2023.01142

4. Schloemann DT, Sajda T, Ricciardi BF, Thirukumaran CP. Association of Total Knee Replacement Removal From the Inpatient-Only List With Outpatient Surgery Utilization and Outcomes in Medicare Patients. *JAMA network open*. Jun 1 2023;6(6):e2316769. doi:10.1001/jamanetworkopen.2023.16769

© 2025 Thirukumaran CP et al. *JAMA Network Open*
