# Supplementary material for: Opioid Cap Laws and Opioid Prescriptions After Total Joint Replacements in Older Adults
Source: JAMA Netw Open. 2025 Apr 9;8(4):e254448. doi: 10.1001/jamanetworkopen.2025.4448 (PMC11983234; doi:10.1001/jamanetworkopen.2025.4448)
Supplement: Supplement 2. — Data Sharing Statement [file jamanetwopen-e254448-s002.pdf]

Data Sharing Statement

Thirukumaran. Opioid Cap Laws and Opioid Prescriptions After Total Joint Replacements in Older Adults. *JAMA Netw Open*. Published April 09, 2025.
doi:10.1001/jamanetworkopen.2025.4448

Data
Data available: No

Additional Information
Explanation for why data not available: We are not allowed to share the data per our agreement with CMS.
